# Forensic Analysis of Synthetic Cathinones on Nanomaterials-Based Platforms: Chemometric-Assisted Voltametric and UPLC-MS/MS Investigation

**DOI:** 10.3390/nano13172393

**Published:** 2023-08-22

**Authors:** Ana-Maria Dragan, Bogdan George Feier, Mihaela Tertiș, Ede Bodoki, Florina Truta, Maria-Georgia Ștefan, Béla Kiss, Filip Van Durme, Karolien De Wael, Radu Oprean, Cecilia Cristea

**Affiliations:** 1 Department of Analytical Chemistry, Faculty of Pharmacy, ‘Iuliu Hațieganu’ University of Medicine and Pharmacy, Pasteur 6, 400349 Cluj-Napoca, Romania; ana.dragan@umfcluj.ro (A.-M.D.); feier.george@umfcluj.ro (B.G.F.); mihaela.tertis@umfcluj.ro (M.T.); florina.truta@umfcluj.ro (F.T.); roprean@umfcluj.ro (R.O.); 2 A-Sense Lab, University of Antwerp, Groenenborgerlaan 171, 2010 Antwerp, Belgium; karolien.dewael@uantwerpen.be; 3 Department of Toxicology, Faculty of Pharmacy, ‘Iuliu Hațieganu’ University of Medicine and Pharmacy, Pasteur 6, 400349 Cluj-Napoca, Romania; stefan.georgia@umfcluj.ro (M.-G.Ș.); kbela@umfcluj.ro (B.K.); 4 Drugs and Toxicology Department, National Institute for Criminalistics and Criminology (NICC), Vilvoordsesteenweg 100, 1120 Brussels, Belgium; filip.vandurme@just.fgov.be; 5 NANOlab Center of Excellence, University of Antwerp, Groenenborgerlaan 171, 2010 Antwerp, Belgium

**Keywords:** electrochemical profile, synthetic cathinones, redox pathway, on-site forensic analysis, chemometric analysis, mass spectrometry

## Abstract

Synthetic cathinones (SCs) are a group of new psychoactive substances often referred to as “legal highs” or “bath salts”, being characterized by a dynamic change, new compounds continuously emerging on the market. This creates a lack of fast screening tests, making SCs a constant concern for law enforcement agencies. Herein, we present a fast and simple method for the detection of four SCs (alpha-pyrrolidinovalerophenone, *N*-ethylhexedrone, 4-chloroethcathinone, and 3-chloromethcathinone) based on their electrochemical profiles in a decentralized manner. In this regard, the voltametric characterization of the SCs was performed by cyclic and square wave voltammetry. The elucidation of the SCs redox pathways was successfully achieved using liquid chromatography coupled to (tandem) mass spectrometry. For the rational identification of the ideal experimental conditions, chemometric data processing was employed, considering two critical qualitative and quantitative variables: the type of the electrochemical platform and the pH of the electrolyte. The analytical figures of merit were determined on standard working solutions using the optimized method, which exhibited wide linear ranges and LODs suitable for confiscated sample screening. Finally, the performance of the method was evaluated on real confiscated samples, the resulting validation parameters being similar to those obtained with another portable device (i.e., Raman spectrometer).

## 1. Introduction

Illicit drug production, trafficking, and abuse are still on the rise, without any sign that this trend will change soon, despite the continuous efforts made by law enforcement agencies (LEAs). An important issue is that the trafficking and consumption of illicit drugs are associated with health hazards, accidents, and criminal acts. In fact, more and more new illicit compounds have been emerging every year with the same or similar parent structures, and the list of illicit drugs is constantly updated. An important example in this regard is represented by synthetic cathinones (SCs), an emerging class of new psychoactive substances (NPSs). A trend of replacing drugs of natural origin (such as cocaine) or semi-synthetic/synthetic ones (such as MDMA) and other popular drugs with SCs has been observed in recent years [1]. SCs are structurally related to the psychostimulant alkaloid cathinone, the natural compound found in *Catha edulis*. These illicit substances are most often sold on the market as powders, frequently referred to as “legal highs” (due to their initial legal status) or labeled as “plant food”, “bath salts”, or “research chemicals”, next to which a printed warning can be found, mentioning that they are “not for human consumption” [2,3].

From a pharmacological point of view, there is a lack of detailed description of the SCs’ effects, although they were described as possessing amphetamine- and cocaine-like effects. Hence, SCs are often sold instead of such traditional drugs of abuse or are used for their adulteration [2,4]. These effects include both desired effects, such as euphoria, hallucinations, and decreased fatigue, as well as many adverse effects, such as cardiovascular anomalies, memory loss, paranoia, or neurotoxicity, to name only a few. Importantly, SCs are a worldwide concern. This group of illicit drugs is characterized by a dynamic change, new compounds with only slight differences in their structures continuously appearing on the market [5]. Globally, SCs were seized mostly in Eastern countries of the EU, Central Asia, and Transcaucasia [6]. In the EU, SCs comprise the second most frequently seized class of NPSs after synthetic cannabinoids, but quantity wise SCs were the highest, accounting for 65% of all materials seized in 2020, according to the most recent EU Drug Report [5]. The fast and constant emergence of new SCs (70 newly emerging NPSs in 2020) accompanied by the market exit of some known SCs (193 NPSs not reported since 2017) [6] grants a dynamic nature to this market, leading to a lack of screening tests [2].

Accordingly, the need for fast, accurate, and portable devices for the screening of suspicious samples fueled the exploration of different analytical methods as potential candidates for the forensic analysis of SCs. Numerous techniques have been applied so far for the identification of illicit drugs in suspected cargo. Among them are gas chromatography coupled with (tandem) mass spectrometry—GC–MS(/MS), most frequently the gold-standard technique, high-performance liquid chromatography with (tandem) mass spectrometry—HPLC-MS(/MS), ion mobility spectrometry (IMS), capillary electrophoresis (CE), and immunoassays [7]. Unfortunately, these techniques present some disadvantages, like high costs, complicated operations, and lengthy analysis time [7,8]. Luckily, many commercial portable devices have been reported for the fast on-site screening of suspected samples based on various methods, such as LC/GC-MS [9], Raman Spectroscopy [10,11,12,13], IMS [14], FTIR spectroscopy [15,16] and colorimetry [17]. Nevertheless, excepting the LC/GC-MS-based devices, these are presumptive tests and often lack selectivity, giving a high number of false negative and false positive results. This is even more the case for SCs which do not induce a color change when the Marquis field test is used, but give false positive reactions with the immunoassay used for methamphetamine [18]. The Zimmermann reagent is the color test recommended by the United Nations Office on Drugs and Crime (UNODC) for generally detecting synthetic cathinones in suspicious samples [19]. However, the colorimetric tests are presumptive tests, lacking specificity and being prone to misinterpretation [7]. Other decentralized methods available are the Raman and FTIR portable devices, which can provide spectral information on the analyzed sample. However, the use of these devices is limited by their high cost, and the analysis of some samples (e.g., liquid or colored samples) can be challenging [7].

Electrochemical methods, on the other hand, are an emerging alternative that offers a fast, portable, low-cost, and accurate analysis of forensic drugs and their metabolites [7,20,21,22]. Therefore, the electrochemical profiles of several SCs, have been reported in the last few years: mephedrone, ethcathinone, methylone, butylone, eutylone [23], MDPV [24], and 4-chloro-alpha-pyrrolidinovalerophenone [23,25]. Moreover, the composition of the confiscated seizures may include other substances such as adulterants/cutting agents (e.g., caffeine, lidocaine, benzocaine) or other illicit drugs (e.g., mixtures of SCs and amphetamines) [26,27,28,29]. These aspects lead to the necessity of constant development of new analytical methods that the LEAs can employ for the detection of this class of illicit drugs. 

In the attempt to expand the knowledge regarding the detection of SCs, the present study aimed the development of a fast and simple method for the detection of four SCs based on their electrochemical profiles in a decentralized manner in suspicious samples (e.g., powders, oils, liquids, or tablets) encountered by the law enforcement agencies in the field (e.g., on the street, at music festivals, in ports or airports). The chosen analytes were *N*-ethylhexedrone (NEH), which accounted for a third of all SCs seized in 2020, 3-chloromethcathinone (3CMC), which accounted for a quarter of all SCs seized in 2020 [5], 4-chloroethcathinone (4CEC), and alpha-pyrrolidinovalerophenone (PVP). In this regard, the electrochemical characterization of the analytes by means of cyclic voltammetry (CV) and square wave voltammetry (SWV) on screen-printed electrodes (SPEs) was explored, and the elucidation of their oxidative pathways by UPLC-MS/MS was performed. Additionally, various chemometric tools (experimental design for screening and optimization, as well as multivariate data analysis) were employed for the rational prediction of the optimal experimental conditions as well as to better understand the critical factors influencing the selectivity and sensitivity of the electrochemical method. Two critical qualitative and quantitative variables were considered: the type of the electrochemical platform (graphite—G, graphene—GPH or multiwalled carbon nanotubes—MWCNT) and the pH of the electrolyte (PBS of pH 7, pH 9.5, and pH 12), respectively. Furthermore, the potentially overlapping electrochemical signals of selected adulterants (procaine—PRO, levamisole—LMS, caffeine—CAF, and creatine—CRE) were also integrated. Thereafter, the analytical figures of merit, such as the linear domain, limit of detection (LOD), and limit of quantification (LOQ), were determined for each analyte. Finally, the method was employed for the screening of the mentioned drugs in confiscated samples. 

Overall, the present study contributes to the expansion of the knowledge regarding SCs detection through various findings which, to the best of our knowledge, were first reported here. Among these is the chemometric-assisted investigation of the voltammetric behavior of the selected analytes at various pH values and on different nanomaterial-based SPEs. Additionally, the electrochemical oxidation mechanisms of the four SCs studied in this work (PVP, NEH, 4CEC, and 3CMC) are also reported.

## 2. Materials and Methods

### 2.1. Materials and Instrumentation

All chemicals used in this study were of analytical grade and were used as purchased from the manufacturers. All solutions used were prepared with ultrapure water (obtained with Adrona B30 water purification system). Standards of 3CMC, 4CEC, NEH, and PVP were purchased from Cayman Chemicals. Standards of caffeine, creatine, acetic acid, dibasic phosphate (K_2_HPO_4_), hydrochloric acid (HCl), levamisole, lidocaine, monobasic phosphate (KH_2_PO_4_), potassium chloride (KCl), and sodium hydroxide (NaOH) were purchased from Merck. The supporting electrolyte used for all electrochemical experiments (except when chronoamperometry was employed) was 100 mM phosphate buffer saline with 100 mM KCl (PBS), which was prepared using K_2_HPO_4_ and KH_2_PO_4_. The pH of the buffer was adjusted with either NaOH or HCl.

Stock solutions (10 mM) were prepared in ultrapure water for all molecules. From these stock solutions, test solutions of specified concentration for each analyte were prepared before each test by dilution with 100 mM PBS, and 50 µL of the solution was added to an SPE for analysis. 

Confiscated samples were provided by NICC (National Institute of Criminalistics and Criminology), Bruxelles, Belgium. The qualitative and quantitative assessments of the confiscated samples were performed using gas chromatography-mass spectrometry (GC-MS) and gas chromatography with a flame-ionization detector (GC-FID), respectively. The applied chromatographic methods are ISO17025 [30] accredited and are continuously evaluated through participation in international quality control programs (United Nations Office on Drugs and Crime—UNODC, and the European Network of Forensic Science Institutes—ENFSI).

The lab-setting electrochemical experiments were performed using an AUTOLAB PGSTAT 302N potentiostat (EcoChemie, Utrecht, The Netherlands) equipped with the associated NOVA 1.10 software, while a portable potentiostat (EmstatBlue, PalmSens, Houten, The Netherlands) was used for the interrogation of the confiscated samples. For comparison, a handheld Raman spectrometer (Bruker Bravo, Bruker Optik GmbH, Ettlingen, Germany) equipped with the TicTac library was used for decentralized analysis of the confiscated samples.

For all electrochemical experiments, custom-made disposable screen-printed electrodes (SPEs) were provided by Metrohm (Madrid, Spain) with a silver (pseudo) reference, a carbon counter electrode, and with graphite working electrodes modified with either graphene (GPH-SPEs) or with multiwalled carbon nanotubes (MWCNT-SPEs), with a 3 mm diameter. 

Data analysis and figure generation were performed using the Origin 8.5 software (OriginLab, Northampton, MA, USA). For better visualization, all the square wave voltammograms (SWVs) presented here were baseline-corrected using the moving average filter included in the NOVA 1.10 software (window size 1) or the PSTrace 5.9 software without affecting the results.

All chemical structures were generated using the free online ChemDraw tool (PerkinElmer Informatics, Hongkong).

### 2.2. Electrochemical Procedures

SVW and CV were used for the investigation of the electrochemical response of the molecules investigated in this study. The SWV parameters were a potential range from 0.0 V to 1.3 V, with a step potential of 5 mV, a scan rate of 50 mV s^−1^, an amplitude of 25 mV, and a frequency of 10 Hz. The CV parameters were: potential window from −0.5 V to 1.3 V, with a step potential of 5 mV and a scan rate of 100 mV s^−1^.

Chronoamperometry (CA) was applied for the electrochemical degradation of the analytes in 10 mM ammonium acetate buffer (AAB) pH 7. This electrolyte was chosen only for this part of the study due to its compatibility with the UPLC-MS/MS system used for the analysis of the solutions that resulted after the electrolysis. The CA parameters were an interval time of 1 point s^−1^ and duration of 30 min and 60 min. The potential applied during CA degradation was set according to the peak potential (*E_p_*) observed on the square wave voltammogram for each SC. 

### 2.3. UPLC-PDA and UPLC-MS(/MS) Procedures

The chromatographic system consisted of a Waters Acquity UPLC system coupled with a Waters PDA detector and a Waters TQD mass spectrometer (Waters, Milford, MA, USA). To obtain MS and MS/MS spectra using direct infusion into the mass spectrometer, the TQD was operated in ESI+ mode, using the following settings: source temperature 150 °C, desolvation temperature 350 °C, cone gas flow 50 L h^−1^, desolvation gas flow 700 L h^−1^, collision gas flow 0.17 mL min^−1^, capillary voltage 3.00 kV. 

All infused solutions had a concentration of 5 μM and were obtained by serial dilutions of stock solutions in a mixture of mobile phase A (5 mM ammonium acetate in water, containing 0.1% formic acid) and B (0.1% formic acid in methanol) in a ratio of A:B 90:10 *v*/*v*. The solutions were infused in the mass spectrometer using the combined mode and an infusion flow of 10 μL min^−1^. Optimum ionization and fragmentation parameters (cone and collision energy, Table S1) were determined by using the Intellistart function of the MassLynx 4.2 software. MS and MS/MS spectra were then recorded using the optimal cone and collision energy for each compound (Table S1). When optimization of ionization and fragmentation conditions using Intellistart was not possible (e.g., when the concentration of an oxidation product was too low), MS and MS/MS spectra were obtained exclusively after chromatographic separation of compounds, with data collection in MS scan mode. Cone energy ramps and collision energy ramps were used to ensure satisfactory ionization and fragmentation for all separated compounds.

Chromatographic separation was achieved by using an XBridge BEH Phenyl Column (130 Å, 3.5 µm, 4.6 mm × 100 mm; Waters, Milford, MA, USA) and gradient elution (Table S2) at a flow rate of 0.5 mL min^−1^. The mobile phase consisted of a mixture of 5 mM ammonium acetate in water, containing 0.1% formic acid (A) and 0.1% formic acid in methanol (B). The samples were kept at 4 °C in the autosampler, and the chromatographic column was maintained at 40 °C. The mass spectrometer was operated in ESI+ mode using the same settings. When monitoring exclusively for the parent compounds, the Selected Ion Recording (SIR) operating mode was used. To identify possible oxidation compounds present in the solution and to obtain MS spectra of such compounds, the MS Scan operating mode was employed. In order to obtain MS/MS spectra of the identified compounds, the mass spectrometer was operated in Daughter Scan mode. Regarding PDA detection, the eluent absorbance was monitored in the 200–300 nm wavelength domain. All presented UPLC-PDA chromatograms were extracted for λ = 250 nm.

### 2.4. Chemometric Analysis

Initially, to identify the most critical experimental variables leading to the most sensitive and potentially the most selective electrochemical detection of the four SCs, a fast-screening study was performed on the binary mixtures of the SCs with some of the most common adulterants. A reduced combinatorial design (13 runs, 3 center points, *n* = 32) with a balanced distribution of all factor settings has been employed, considering as responses the current intensities of the SCs (*I_p_* SC) and adulterants (*I_p_* A) as well as the recorded separation between the peaks of the SCs and the adulterants (d*E_p_*(SC-A)) recorded by SWV. Additionally, for a more thorough understanding of the variables’ influence and a more accurate prediction of the ideal experimental conditions, a full factorial optimization design was performed on the recorded SWV data using 0.5 mM solutions of each SC and each adulterant. Linear models using PLS have been fitted to the data (4 variables, 3 responses—the recorded separation between the peaks of the SCs among themselves and the adulterants—d*E_p_*(SC-I), the current intensities of the SCs—SCs—*I_p_* SCs, the current intensities of the interferants—*I_p_* I) using Modde Pro v.13.0.1 (Sartorius Stedim Data Analytics AB, Sweden). Besides the tested SCs, three other quantitative and qualitative variables were also considered: (i) a multilevel quantitative variable: the pH of the electrolyte at values of 7, 9.5, and 12, and (ii) two qualitative variables: (a) the platform type including G-SPEs, and GPH-SPEs and MWCNT-SPEs, and (b) the presence of adulterants (PRO, LMS, CAF, and CRE) in equimolar binary mixtures (0.5 mM:0.5 mM) with each of the four SCs for the screening study, and in single solutions for the optimization study. Each experiment was performed in triplicate. Additional data mining has been performed on the same data set by multivariate regression analysis (OPLS) between the predictors (different experimental variables) and response variables scaled to unit variance using Simca 17 (Sartorius Stedim Data Analytics AB, Umeå, Sweden).

### 2.5. Analytical Performance

The analytical performance of the method was evaluated by testing 50 µL solutions of increasing concentrations (1, 5, 10, 25, 50, 75, 100, 150, 250, 500, 750, and 1000 µM) by SWV in optimized conditions of pH and platform composition. The obtained oxidation currents were plotted against the corresponding concentrations to build the calibration curves, based on which the LOQ and LOD were determined as the lower limit of the calibration curve and as 1/3 × LOQ, respectively. All tests were performed in triplicate.

### 2.6. Confiscated Samples Analysis

A disposable spatula was used for the sampling of the confiscated samples for their dissolution/dilution in the buffer of appropriate pH (approximately 1 mg in 15 mL). Moreover, 50 µL of the obtained solutions were then interrogated on the disposable SPEs inserted into the portable potentiostat connected to a laptop through Bluetooth. The sensitivity, specificity, and accuracy were determined as frequently encountered parameters for the validation of portable methods for illicit drug screening [31,32,33,34,35] to validate the relevance of the method. The following formulas were applied:

sensitivity = TP/(TP + FN) × 100,(1)

specificity = TN/(TN + FP) × 100(2)

accuracy = (TP + TN)/(TP + TN + FP + FN) × 100(3)

where TP: true positive, TN: true negative, FP: false positive, and FN: false negative.

## 3. Results

### 3.1. Electrochemical Behavior of the Investigated Molecules

**Figure 1:**
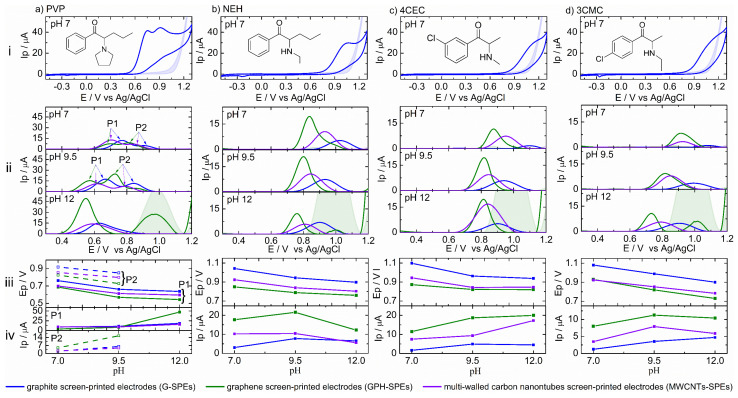
Electrochemical characterization of the four synthetic cathinones (SCs): (**a**) PVP, (**b**) NEH, (**c**) 4CEC, and (**d**) 3CMC, on G-SPE (blue), GPH-SPE (green), and MWCNT-SPE (purple) in 0.5 mM solutions in PBS 20 mM at pH 7, pH 9.5, and pH 12: (**i**) cyclic voltammograms (at pH 7) with the corresponding structures of the SCs, (**ii**) baseline corrected square wave voltammograms; (**iii**) the shift of SCs peak potential (*E_p_*) and (**iv**) current intensity (*I_p_*) with the pH of the electrolyte. The blue/green/purple filled areas represent the blanks on the G-SPEs, GPH-SPEs, and MWCNT-SPEs, respectively. P1 and P2: the two oxidation peaks registered for PVP at pH 7 and pH 9.5.

The investigation by SWV at three different levels of pH (7, 9.5, and 12, chosen based on previous studies [23,35]) and on three different platforms (G-SPE, GPH-SPE, and MWCNT-SPE) revealed that all tested molecules are electroactive in all testing conditions, except CAF and CRE for which no signal was registered at all. The characterization of the four SCs at pH 7 on G-SPEs by CV (Figure 1i) revealed the irreversible nature of the electrochemical oxidation of PVP, NEH, 4CEC, and 3CMC at 0.76 V/0.92 V (a split peak), 1.04 V, 1.10 V, 1.08 V, respectively. The SWV analysis (Figure 1ii) allowed the determination of the *E_p_* values for the oxidation of each SC in all conditions tested: between 0.54 V and 0.92 V for PVP, 0.77 V and 1.04 for NEH, 0.82 V, and 1.10 V, and 0.73 V and 1.08 V (Table S3). These values were used to establish the working potential applied during CA for the electrochemical degradation of each SC for the redox pathway elucidation on G-SPEs.

Regarding the influence exerted by the nature of the platform, a cathodic shift of the oxidation *E_p_* (Figure 1iii) and an increase of the *I_p_* (Figure 1iv) were observed when the nanomaterial-based platforms were used, a higher effect was registered for the GPH-SPEs than for the MWCNT-SPEs. Hence, the *E_p_* values decreased, and the *I_p_* values increased, in the following order: G-SPEs, MWCNT-SPEs, GPH-SPEs. These influences may be attributed to the higher surface available for electron transfer provided by the nanomaterials on the SPEs [36,37]. 

However, a few deviations from the general influences mentioned earlier were observed: (i) the *I_p_* registered for P1 of PVP at pH 7, the lowest value being registered on the GPH-SPEs, at pH 9.5, similar values being obtained on all platforms, and pH 12, similar values being registered on the G-SPEs and MWCNT-SPEs (Figure 1iv(a)); (ii) the *I_p_* registered for P2 of PVP, similar values being obtained on the G-SPEs and MWCNT-SPEs (Figure 1iv(a)); (iii) the *I_p_* of NEH at pH 12, the lowest value being registered on the MWNCNT-SPEs (Figure 1iv(b)); (iv) the *E_p_* of 3CMC at pH 7, similar values being obtained on the GPH-SPEs and MWCNT-SPEs (Figure 1iv(d)).

Similar influences were generated by the pH of the electrolyte. For all the analytes, a cathodic shift of the oxidation *E_p_* (Figure 1iii) was observed with the increase of the pH of the electrolyte on all platforms, except for 4CEC on GPH-SPEs and MWCNT-SPEs for the increase of pH from 9.5 to 12 (the *E_p_* being similar at both pH values; Figure 1iii(c)). The *I_p_* (Figure 1iv) varied differently among the four SCs. For PVP, an increase of the *I_p_* was observed with the increase of the pH on all platforms, but it was more significant on the GPH-SPEs (Figure 1iv(a)). It is worth mentioning that for PVP, a change of the peak shape was also observed, from an incompletely split peak at pH 7 and pH 9.5 to a single peak at pH 12, contributing to the *E_p_* and *I_p_* changes. This is probably due to the alkaline media, which facilitates the electrochemical oxidation of PVP, the two consecutive reactions involved in the electrochemical oxidation of PVP (as shown in the redox-pathways elucidation of this SC) happening simultaneously at pH 12. This particularity can be translated into a more characteristic signal. For NEH, 4CEC, and 3CMC, the *I_p_* increased with the variation of the pH from 7 to 9.5 on all platforms (Figure 1iv(b–d), respectively). The further increasing of the pH to 12 determined variable effects on the *I_p_*: (i) no significant effects for NEH on G-PSEs, and for 4CEC on G-SPEs and GPH-SPEs (Figure 1iv(b,c), respectively); (ii) an increase for 4CEC on MWCNT-SPEs, and for 3CMC on G-SPEs (Figure 1iv(c,d), respectively); (iii) a decrease for NEH and 3CMC on the nanomaterial-based SPEs (Figure 1iv(b,d), respectively). The described influences suggest the involvement of protons in the oxidation mechanism of the four SCs, which was further confirmed when the elucidation of the redox pathways was performed (see corresponding section). In any case, a lower oxidation potential is beneficial for electrochemical detection since the risk of interferents decreases as well. The relationships between the *E_p_* and pH for all SCs and on all platforms are described by the equations in Table 1. 

On all platforms, the four SCs exhibited approximately half of the Nernstian slope (i.e., 0.059 V pH^−1^ at 25 °C), indicating a 1:2 ratio for the protons and electrons involved in their oxidation mechanism [23].

As previously mentioned, from the four adulterants tested, an electrochemical response was obtained only for PRO and LMS, which presented oxidation peaks in all experimental conditions (Figure S1i). For PRO, the recorded oxidation peaks were in the window of potential from 0.53 V to 0.83 V (Table S4), overlapping with the oxidation peaks observed for PVP. This is not surprising since both molecules contain a tertiary amine in their structures (Figure S2 and Figure 1i(a), respectively). 

Moreover, the same effects induced by the increase of the pH and by the presence of nanomaterials on the signal of SCs were also observed for PRO (a cathodic shift of the *E_p_*—Figure S1ii(a), and an increase in *I_p_*—Figure S1iii(a)). However, on the GPH-SPEs, a change of the peak shape was observed with the increase of pH from 7 to 12: from a single peak to a split peak (in opposition to the change in shape observed for PVP), which could also explain the lower *I_p_* on this platform at pH 12 compared to pH 9.5. This particularity observed for both molecules at pH 12 on the GPH-SPEs can be exploited for the elimination of the only overlap in signal observed in this study, namely the one between PVP and PRO.

The oxidation peaks of LMS were obtained at slightly higher potential values than those of all four SCs for each fixed combination of parameters (in the potential window from 0.97 V to 1.2 V; Table S4). As in the case of the SCs, a slight cathodic shift of the oxidation *E_p_* was observed with the increase of pH from 7 to 12, with a more pronounced shift generated by the presence of the carbon nanomaterials on the surface of the platform (Figure S1ii(b)). On the other hand, the increase of the electrolyte pH caused an increase of the *I_p_*, while the presence of the carbon nanomaterials had different effects: MWCNT caused a slight increase, while GPH caused a decrease of the signal intensity (Figure S1iii(b)). Given the obtained results, the investigated adulterants/cutting agents could generate some challenges for the detection of the SCs when mixtures would be analyzed, given the possible overlapping of PRO signal and PVP or the close values of the LMS and NEH, 4CEC, 3CMC oxidation potentials. Moreover, the simultaneous detection of NEH, 4CEC, and 3CMC might prove challenging, given the close values of their oxidation potentials. Therefore, applying the univariate visual approach for the selection of the optimal pH and platform, both the *I_p_* and the oxidation *E_p_* were considered, as well as the electrochemical response of the adulterants. Consequently, given the fact that PVP and 4CEC showed the highest current intensities at pH 12, while NEH and 3CMC at pH 9.5, pH 12 was considered optimal since at this pH, the values of the oxidation *E_p_* were the lowest for all SCs and the difference between them was the highest. Regarding the platform, GPH-SPEs were considered optimal since on this electrode surface, the lowest oxidation *E_p_* and the highest current intensities were recorded, with one exception: the oxidation *E_p_* of 3CMC on MWCNT-SPEs, but at pH 7, not pH 12. Moreover, at pH 12 on the GPH-SPEs, none of the studied adulterants should hinder the detection of the four SCs. An overlap of the SWVs obtained for the SCs and the adulterants is shown in Figure S3.

Additionally, the reproducibility was evaluated in all experimental conditions for all SCs and all adulterants. Importantly, for all the SCs, better results were obtained on the nanomaterial-modified SPEs. For *E_p_*, the obtained values varied in a very narrow range, 0.22–3.0%, 0.0–2.1%, and 0.2–1.2% for the G-SPEs, GPH-SPEs, and MWCNT-SPEs, respectively. In the case of *I_p_*, the RSDs obtained were 2.61–14.98%, 1.62–15.92%, and 0.72–16.59% for the G-SPEs, GPH-SPEs, and MWCNT-SPEs, respectively. Further details on the RSDs obtained for both the SCs and the adulterants can be found in Table S3 and Table S4, respectively. Overall, these values can be considered acceptable since, for the screening of suspected samples, the qualitative analysis, mainly based on peak potential values, is of interest for the LEAs.

### 3.2. Redox Pathways Elucidation

**Figure 2:**
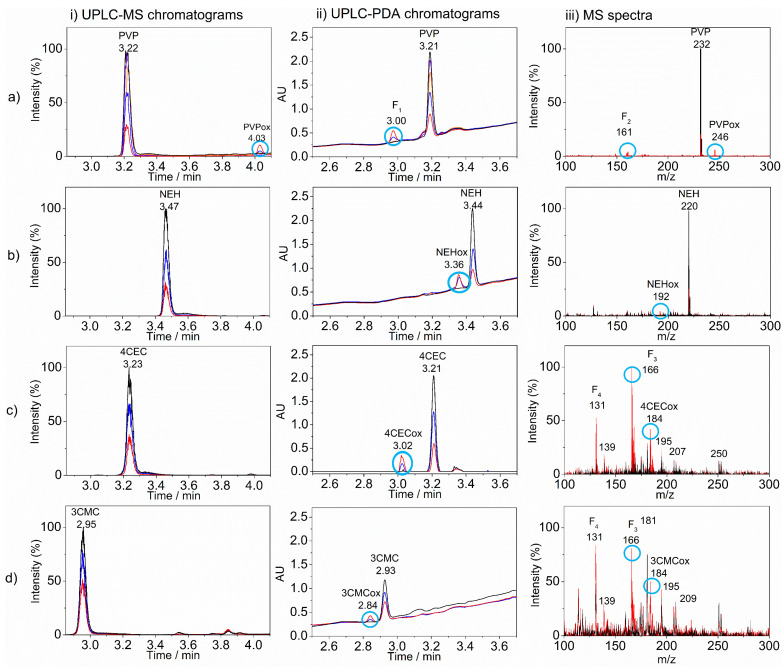
(**i**) UPLC-MS chromatograms, (**ii**) UPLC-PDA chromatograms, and (**iii**) MS spectra of the 1 mM control solutions (black) and the 1 mM electrolyzed solutions for 30 min (blue/purple) and 60 min (red/orange) at (**a**) 1.03 V/0.84 V for α—pyrrolidinovalerophenone (PVP), (**b**) 1.20 V for N-ethylhexedrone (NEH), (**c**) 1.25 V for 4-chloroethcathinone (4CEC) and, (**d**) 3-chloromethcathione (3CMC). The MS spectra for PVP and NEH were obtained by direct infusion of control and electrolyzed solutions in the MS, while in case of 4CEC and 3CMC, the MS spectra were extracted at the retention time corresponding to the oxidation product, following UPLC-PDA-MS analysis; F: retention times attributed to fragments of the oxidation products. Blue circles: supplementary peaks observed for the electrolyzed solutions in comparison to the control solutions.

The electrolysis of all four SCs was confirmed by the decrease of the parent peak on both the UPLC-MS (Figure 2i) and UPLC-PDA (Figure 2ii) chromatograms, as well as on the MS spectra (Figure 2iii) of the electrolyzed solutions in comparison to the control solutions. 

The peak diminishment was positively correlated with the employed electrolysis time for all SCs, as well as the value of the oxidizing potential in the case of PVP, the electrolysis at *E_p_* = 1.03 V (Figure 2a blue and red) being more significant than at *E_p_* = 0.84 V (Figure 2a purple and orange). Furthermore, for the electrolyzed solutions, the MS spectra and the UPLC-MS and UPLC-PDA chromatograms registered new peaks (marked with blue circles in Figure 2) besides the ones attributed to the parent compounds.

**Scheme 1:**
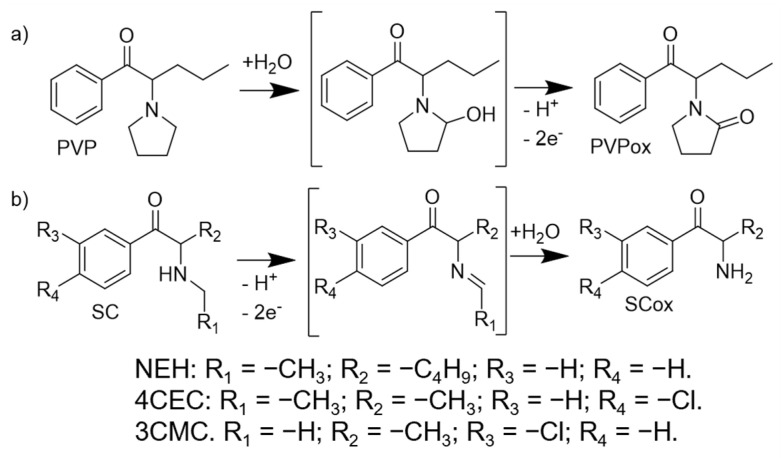
Oxidation mechanisms of (**a**) PVP and (**b**) NEH, 4CEC, and 3CMC based on the UPLC-MS(/MS) data. PVPox and SCox are the oxidized products of PVP and the other three SCs, respectively.

The results obtained for PVP electrolysis indicated the formation of two new peaks, one at *m*/*z* = 161 and one at *m*/*z* = 246. The first one seems to correspond to a fragment of the oxidized compound of PVP (PVP_ox_) since it appears in the MS/MS spectra of this compound as well (Figure S4b(i)). Therefore, the proposed structure of the PVP_ox_ (which is also a known metabolite of PVP [38]) with *m*/*z* = 246 is shown in Scheme 1a. Given the fact that there was a split peak on the SWVs of PVP (Figure 1ii(a)) at pH 7, the electrochemical oxidation likely takes place first through the hydroxylation of the pyrrolidine nucleus, followed by the subsequent oxidation of this group when higher anodic potentials are reached (Scheme 1a), a behavior that has previously been reported for 4-Cl-PVP by Schram et al. [23].

The results obtained for the electrolysis of NEH indicated the formation of one new peak at *m*/*z* = 192, which seems to be the oxidized product of NEH (NEHox) since it only appears in the MS spectra of the electrolyzed solutions (marked with a blue circle on Figure 2iii(b)) and not on the MS spectra of the control solution. Hence, NEH_ox_ is the result of an oxidative dealkylation of the secondary amine that takes place during the electrochemical oxidation of NEH (Scheme 1b).

Similar to PVP, for both 4CEC and 3CMC, there were two supplementary peaks in the MS spectra of the electrolyzed solutions compared to the control solutions: one at *m*/*z* = 184 and one at *m*/*z* = 166 (Figure 2iii(c,d)). The peak at *m*/*z* = 166 seems to result from the compound with *m*/*z* = 184 after the loss of one water molecule, while the *m*/*z* = 184 peak seems to correspond to the oxidized products of 4CEC and 3CMC (4CECox and 3CMCox, respectively), since a peak at *m*/*z* = 184 appeared on the MS spectra of the electrolyzed solutions, but did not appear on the spectra of the control solution (Figure 2iii(c,d)). Hence, the proposed oxidation mechanism of 4CEC and 3CMC is the oxidative dealkylation of the secondary amine (Scheme 1b), just as in the case of NEH, findings that were consistent with the results reported for other SCs [23,39].

Fragmentation patterns (Figures S4–S7) for both the parent molecules and the oxidation products were proposed based on the MS/MS spectra obtained for both types of solutions (control solution, applied on the electrode without applying any potential, and electrolyzed solutions). The fragmentation patterns may contain two main ways of fragmentation based on whether or not there is a first water loss step, which was reported in the literature for certain SCs [39]. The major fragments are summarized in tables for each molecule (Tables S5–S8).

### 3.3. Chemometric Optimization of the Method

A reduced combinatorial screening study on the data obtained from the analysis of the binary mixture solutions in different conditions (Tables S9 and S10) was employed. The objective of the screening study was to reveal the experimental variables providing the best resolution in terms of *E_p_*—d*E_p_*(SC-A), as well as the highest current intensities for the SCs—*I_p_* SCs and the opposite (lowest signals) for the tested adulterants—*I_p_* A (further details on the model objectives can be consulted in Table S11). Hence, a linear PLS model with a fair to excellent measure of fit (R^2^ between 0.84 and 0.98; Figure S8a) was obtained, revealing that a more alkaline pH (9.5 for PVP and NEH, and 12 for 3CMC and 4CEC) and the nanomaterial-based platforms (GPH-SPEs for PVP and NEH, and MWCNT for 4CEC) would offer the best signal to noise ratio for the electrochemical detection of the tested SCs. 

Following the outcomes of the screening design, a full factorial optimization design was performed on the recorded SWV data using equimolar 0.5 mM pairwise combinations of each SC (Table S3) with each adulterant (Table S4) under all experimental conditions (three levels of pH: 7, 9.5, and 12, and three different platforms: G-SPEs, GPH-SPEs, and MWCNT-SPEs). The study aimed to predict the experimental conditions providing the highest detection selectivity, thus maximum d*E_p_*(SC-I) (minimum set value of 100 mV), accompanied by the best analytical sensitivity, thus maximum *I_p_* for SCs, while lowest *I_p_* for the adulterants (further details on the model objectives can be consulted in Table S12). Besides the tested adulterants, each of the studied SCs was also considered as potential interferents (I) in the detection of a given SC. To meet these objectives, a linear PLS model with a fair to good measure of fit (R^2^ between 0.54 and 0.78, Figure S8b) for each response has been obtained. Subsequently, an individual optimization run was performed for each of the studied SC while all the other variables (pH, platform, adulterants) were set free. As such, model predictions revealed that a more alkaline pH (8 for PVP and 12 for the other SCs) using the nanomaterial-based platforms (GPH-SPEs for all SCs) would bring optimal conditions for the SCs detection, also confirming the univariate visual interpretation of the voltametric responses.

Further data analysis revealed that for the average predicted d*E_p_*(SC-I) values, neither the type of the electrode (Figure S8c(i)) nor the pH (Figure S8c(ii)) is detrimental in achieving the minimum set value of 100 mV for this response. However, these parameters are critical in the case of certain interferents, mostly certain pairs of SC, especially between NEH, 4CEC, and 3CMC, for which very close values of the *E_p_* were registered (Figure S8c(iii)).

**Figure 3:**
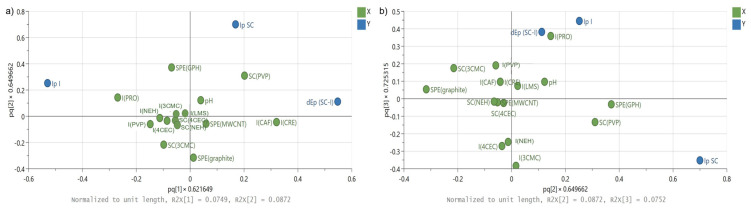
OPLS loadings plots for (**a**) the first two and (**b**) the second and third predictive components showing correlations between the studied factors (X, green) and responses (Y, blue).

For more in-depth data mining, additional multivariate regression analysis using OPLS has been performed on the same experimental data (no. of SWV measurements, *n* = 252) as the training set. The obtained model containing three predictive components showed a good measure of fit and predictivity (R2X(cum) 0.24; R2Y(cum) 0.66; Q2(cum) 0.61). The resulting loadings plot (Figure 3) revealed concealed information about the relationship between the studied factors (X) and response (Y) variables, such as how the studied responses vary in relation to each other, which ones provide similar information, and what is their relationship to the X-variables in the model. The first component of the semiempirical model, accounting for about one-third of the systematic information in the X-block predictive to Y, seems to be simultaneously associated with the selectivity (d*E_p_*(SC-I)) of the SWV measurements and negatively correlated with the recorded *I_p_* of interferents (*I_p_* I), whereas the second component, of a similar predictive contribution (1/3rd), is associated with the detection sensitivity (*I_p_* SC) of the SWV measurements towards the target analytes (Figure 3a). 

Amongst the tested adulterants, CAF and CRE are less likely to affect the selectivity of the electrochemical detection of SCs, regardless of the used electrochemical platform, while PRO is generating the highest signal (*I_p_* I) amongst the tested interferents, being also negatively correlated with the selectivity of detection (d*E_p_*(SC-I)). The overall sensitivity of the electrochemical detection is positively correlated with the GPH-SPE, the highest being for PVP, whereas the lowest signals are being achieved on G-SPE, 3CMC providing the lowest recorded current intensities within the tested experimental conditions. The poorest contributions to the investigated responses are given by the factors lying around the origin of the plot, including pH or MWCNT-SPE, which show modest to low overall contributions for both detection selectivity and sensitivity. Similar correlations are to be seen in the loadings plot for the second and third (mainly associated with the recorded signal-to-noise ratio induced by the potential interference of the adulterants) predictive component of the model (Figure 3b). 

### 3.4. Analytical Performance of the Method

For the qualitative analysis, windows of potential for the SCs detection were defined based on the *E_p_* obtained during the analytical evaluation to ensure the allocation of each SCs in its own detection window on the entire concentration range studied. Hence, the detection windows (DWs) were 0.47–0.57 V (DW1), 0.70–0.79 V, 0.74–0.86 V, and 0.69–0.76 for PVP, NEH, 4CEC, and 3CMC, respectively. An overlap of the NEH, 4CEC, and 3CMC detection windows was thus obtained, which is not surprising given their very similar structures (Figure 1i) and their oxidation mechanism (Scheme 1). Hence, if a peak is registered in the window of potential DW1, the test would be positive for PVP, and if a peak is obtained between 0.69 V and 0.86 V (DW2), the test would be positive for one of the other three SCs, additional tests being required to aid the precise identification of the SC (e.g., complementary methods such as Raman or FTIR spectroscopy, or confirmatory analysis by GC-MS). Nevertheless, the described strategy provides a fast and user-friendly way for the LEAs to detect these SCs in a suspicious sample regardless of its nature (e.g., liquid, pastes, oils, powders) or its color, thus aiding the decision regarding the analyzed sample. These are important advantages since other portable instruments (e.g., the Raman device) may present difficulties when analyzing liquid or colored samples [7].

**Figure 4:**
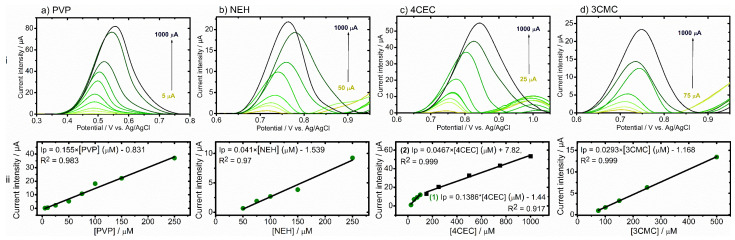
Baseline-corrected SWVs (**i**) registered at pH 12 on GPH-SPEs in the 1–1000 µM range, with the obtained linear curves and the corresponding equations (**ii**) of (**a**) PVP in the 5–250 µM domain, (**b**) NEH in the 50–250 µM domain, (**c**) 4CEC in the 25–100 µM (1), and 100–1000 µM (2) domains, and (**d**) 3CMC in the 75–500 µM domain.

The analytical performance evaluation further revealed a linear increase in the peak height with respect to the concentration for all four SCs in various concentration ranges (Figure 4) with LODs of 1.67 µM, 16.67 µM, 8.33 µM, and 25 µM for PVP, NEH, 4 CEC, and 3CMC, respectively.

Compared to the method described herein, some electrochemical detection methods previously reported (Table 2) were characterized by lower LODs. On the other hand, these methods employed more complex platforms or were accompanied by a pre-treatment step using separation or extraction techniques. This can be justified in the case of clinical applications (as was the case of the referred papers), whilst the analysis of major constituents in confiscated samples can be comfortably achieved with LODs in the µM range. It is worth mentioning that, at first sight, some authors reported a theoretical LOD, which proved to be lower than the lowest measurable signal, which would rather be the relevant value in-field [40]. Therefore, we are confident that the method described herein, using a simpler, mass-produced, cost-effective, and more robust electrochemical platform, is suitable for the on-site screening of suspicious samples.

### 3.5. Confiscated Samples Analysis

The GC-MS analysis (Table S13) revealed nine samples containing SCs (S1–S9), six containing other illicit drugs (S10–S15), and six containing only adulterants (S16–S21). For the samples containing SCs (S1–S9), the GC-MS analysis did not indicate the presence of any other compound besides the indicated SC. Therefore, these samples were not adulterated. Regarding the samples containing other illicit substances (S10–S15), a few of them were adulterated (S10, S12, S13, and S14), and the adulteration ratio indicated by the GC-FID analysis was between 3% and 65%. 

**Figure 5:**
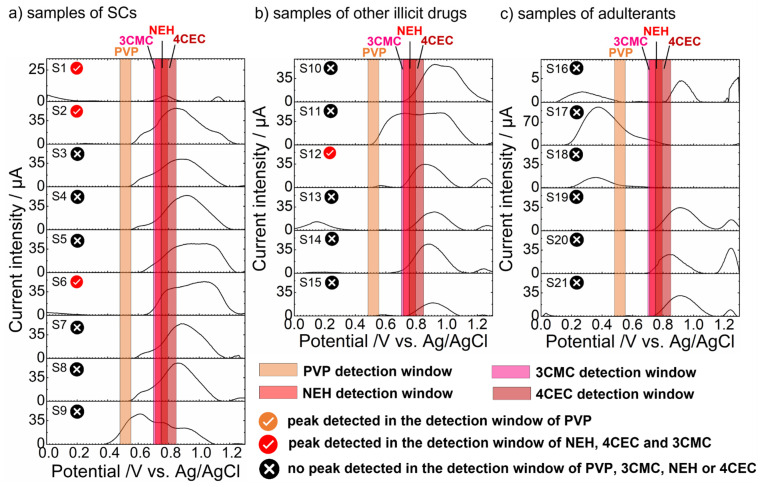
Baseline corrected square wave voltammograms (SWVs) of the seized samples analyzed in PBS pH 12 on graphene screen printed electrodes (GPH-SPEs). (**a**) Samples 1–11 containing synthetic cathinones (SCs), (**b**) samples 12–17 containing other illicit drugs, and (**c**) samples 18–23 containing only adulterants. Table S13 contains a list of the identified compounds in the samples by the standard method.

Finally, the optimized electrochemical method was employed for the screening of 21 confiscated samples containing SCs, other illicit drugs, or adulterants. The registered oxidation peaks (Table S13) on the obtained SWVs (Figure 5) were compared with the defined windows of detection to assess the presence of one of the four SCs. 

An electrochemical test was labeled as positive if the oxidation peak would fit the DW1 or DW2, otherwise being labeled negative. For the Raman spectroscopic measurements (Table S13), a sample was labeled as positive if the displayed result would correspond to any SC or illicit drug and negative if it displayed any other compounds. For both portable instruments, the tests were labeled “true” or “false” by comparison with the results obtained by GC-MS (Table S13). For the electrochemical device, the “true” label was attributed if the correct SC (PVP or one of the other three) was identified for the positive tests or if no peak was detected in the detection windows defined for the SCs in the case of negative tests. In the case of Raman spectroscopy, the “true” label was attributed if the displayed compound corresponded to the correct class (i.e., illicit drugs or adulterants) by comparison with the GC-MS results.

Thereafter, the sensitivity, specificity, and accuracy were determined (Table 3). As can be observed, the results obtained with the electrochemical method were superior to those obtained with the portable Raman spectrometer in terms of sensitivity and accuracy and inferior in terms of specificity. Nevertheless, these results reflect the important contribution that electrochemical methods could bring to the field of forensic analysis.

## 4. Conclusions

The voltametric characterization by SWV in various experimental conditions revealed that the four SCs studied registered oxidation peaks in the potential range from 0.52 V to 1.05 V. Three of them (NEH, 4CEC, and 3CMC) registered very close values of the *E_p_* due to the similarities in their structure, observations supported by the redox pathways elucidation results.

In this extensive study, the method optimization was aided by chemometric analysis, which revealed GPH-SPEs and alkaline pH as optimal analysis conditions. The optimized method exhibited wide linear ranges, with LODs suitable for the screening of confiscated samples. Knowing the electrochemical behavior of the targets on a wide range of concentrations is important since the content of the suspicious samples could significantly vary on the market, and the targeted analytes can register slight shifts in the electrochemical profile. This fact was further observed by applying the method for the detection of the targets in confiscated samples. The obtained results showed promising outcomes for the implementation of electrochemistry in the field of illicit drug detection.

## Figures and Tables

**Table 1:** The relationship between the peak potential and pH on the three platforms.

Synthetic Cathinone	Platform	Equation	R^2^
PVP (peak 1)	G-SPEs	*E_p_* (V) = −0.025 pH + 0.924	0.783
	GPH-SPEs	*E_p_* (V) = −0.029 pH + 0.872	0.755
	MWCNT-SPEs	*E_p_* (V) = −0.021 pH + 0.834	0.741
NEH	G-SPEs	*E_p_* (V) = −0.029 pH + 1.236	0.920
	GPH-SPEs	*E_p_* (V) = −0.011 pH + 0.938	0.557
	MWCNT-SPEs	*E_p_* (V) = −0.024 pH + 1.086	0.902
4CEC	G-SPEs	*E_p_* (V) = −0.032 pH + 1.302	0.729
	GPH-SPEs	*E_p_* (V) = −0.011 pH + 0.938	0.557
	MWCNT-SPEs	*E_p_* (V) = −0.020 pH + 1.065	0.456
3CMC	G-SPEs	*E_p_* (V) = −0.036 pH + 1.333	0.999
	GPH-SPEs	*E_p_* (V) = −0.040 pH + 1.205	0.992
	MWCNT-SPEs	*E_p_* (V) = −0.028 pH + 1.121	0.999

3CMC: 3-chloromethcathione; 4CEC: 4-chloroethcathinone; *E_p_*: peak potential; G: graphite; GPH: graphene; MWCNTs: multi-walled carbon nanotubes; NEH: *N*-ethylhexedrone; PVP: α-pyrrolidinovalerophenone; SPE: screen-printed electrode.

**Table 2:** Analytical figures of merit for electrochemical methods reported for SCs analysis.

SCs	Platform	Technique	LOD (µM)	Linear Range (µM)	Real Sample	Ref.
4Cl-aPVP	G-SPE	SWV	39.9	50–500	street samples	[41]
4Cl-aPVP	SDS-G-SPE	AdSDPV	1.6	2.5–30	oral fluid	[25]
4F-NEB	CEM	POT	NP	119.57–4128.83	-	[42]
4MEC	GO-MIPs/GCE	DPV	2.29	26.14–313.69	-	[43]
4MEC	G-SPE	HPLC-AMP	48.9	261.51–2615.06	street samples	[44]
4MEC	Bi/Hg-G-SPE	CV	60.67	523.01–2092.05	street samples	[45]
4MEC	Coin	CV	2.61	0.005–0.52	street samples	[46]
4MMC	G-SPE	HPLC-AMP	82.78	282.33–2823.26	street samples	[47]
4MMC	Bi/Hg-G-SPE	CV	66.59	564.33–2257.34	street samples	[45]
4MMC	Coin	CV	3.16	0.006–0.56	street samples	[46]
BUP	TH3CA4AB15C5—PtDE	EIS	NP	20–1220	-	[48]
BUP	CEM	POT	NP	118.51–4221.22	-	[42]
BUTY	G-SPE	SWV	65.3	100–500	street samples	[41]
ETC	G-SPE	SWV	157.2	200–1000	street samples	[41]
EUTY	C-SPE	DPV	2	20–600	street samples	[40]
EUTY	BDDE	SPEX-SWV	3.7	13–88	street samples	[49]
MDPPP	TH3CA4AB15C5–PtDE	EIS	NP	39.9–1220	-	[48]
MDPPP	CEM	POT	NP	12.13–4015.37	-	[42]
MDPV	C-MWCNT-SPE-AgNP-MIP	SWV	1.8	1.8–75	-	[50]
MDPV	MDPV-CBSA-SF/MIL-68@Sn-TCPP/GCE	ECL	5.08 × 10^−16^	3.63 × 10^−15^–3.63 × 10^−9^	e-cigarettes	[51]
MDPV	G-SPE	AdSDPV	0.5	1.6–100	street samples	[24]
MEP	G-SPE	SWV	27.8	100–1000	street samples	[41]
MEP	GPH-SPE	AdSDPV	0.3	2.6–112	-	[52]
METY	G-SPE	SWV	79.2	100–500	street samples	[41]
METY	Au-SPE-MIP	SWV	1.1	3.6–50	urine, serum	[53]
METY	CEM	POT	NP	188.22–2813.71	-	[42]
MEP	MIP/PT/MWCNT@AuNPs GCE	SWV	0.0008	0.001–0.01; 0.01–0.1	urine, plasma	[53]
PVP	GPH-SPEs	SWV	1.67	5–1000	street samples	This work
NEH	GPH-SPEs		16.67	50–250		
4CEC	GPH-SPEs		8.33	25–100; 100–1000		
3CMC	GPH-SPEs		25	75–500		

Targets: 3CMC: 3-chloromethcathinone; 4CEC: 4-chloroethcathinone; 4Cl-aPVP: 4-chlor-alpha-pyrrolidinovalerophenone; 4F-NEB: 4-fluoro-N-ethylbuphedrone; BUP: buphedrone; BUTY: butylone; ETC: ethcathinone; MDPPP: 3′,4′-methylenedioxy-alpha-pyrrolidinopropiophenone; MDPV: 3,4-methylenedioxypyrovalerone; MEP: mephedrone; METY: methylone; NEH: N-ethylhexedrone; PVP: alpha-pyrrolidinovalerophenone. Platforms: Au-SPE-MIP: gold SPE modified with MIP; Bi/Hg-G-SPE: bismuth and mercury film-covered graphite SPE; CME: cation exchanging membrane; G-SPE: graphite SPE; GPH-SPE: graphene SPE; MDPV-CBSA-SF/MIL-68@Sn-TCPP/GCE: glassy carbon electrode modified with cooperative-binding split-aptamers for MPDV loaded in a metalloporphyrin derivative-based metal-organic framework; C-MWCNT-SPE-AgNP-MIP: silver nanoparticles and multiwalled carbon nanotubes covered carbon SPE modified with MIP; GO-MIPs/GCE: glassy carbon electrode modified with graphene oxide and molecularly imprinted polymers nanocomposite; MIP/PT/MWCNT@AuNPs GCE: GCE modified with polytyramine, multiwalled carbon nanotubes and gold nanoparticles functionalized MIP; MIP: molecularly imprinted polymer; SDS-G-SPE: sodium dodecyl sulfate-assisted graphite SPE; SPE: screen-printed electrode; TH3CA4AB15C5-PtDE: platinum disk electrode coated with 4′-(N-3-thiophenecarboxamido)benzo-15-crown-5. Techniques: AdSDPV: adsorptive stripping differential pulse voltammetry; CV: cyclic voltammetry; DPV: differential pulse voltammetry; ECL: electrochemiluminescence; EIS: electrochemical impedance spectroscopy; HPLC-AMP: high-performance liquid chromatography assisted amperometry; POT: potentiometry; SPEX-SWV: solid phase extraction assisted SWV; SWV: square wave voltammetry. Others: LOD: limit of detection; LOQ: limit of quantification; NP: not provided.

**Table 3:** Validation parameters for the synthetic cathinones screening in seized samples by portable methods.

Device	TP	TN	FP	FN	Sensitivity	Specificity	Accuracy
EC reader	2	17	2	0	100.00	89.47	90.48
Raman device	3	6	0	11	21.43	100.00	45.00

EC: electrochemical; FN: false negative; FP: false positive; TN: true negative; TP: true positive.

## Data Availability

Data are contained within the article or Supplementary Material.

## Supplementary Materials

The following supporting information can be downloaded at: https://www.mdpi.com/article/10.3390/nano13172393/s1, Figure S1: Baseline-corrected SWVs of adulterants/cutting agents; Figure S2: Chemical structures of the adulterants/cutting agents; Figure S3: Overlapped SWVs of SCs and adulterants/cutting agents; Figure S4: MS/MS spectra and fragmentation patterns for PVP and PVPox; Figure S5: MS/MS spectra and fragmentation patterns for NEH and NEHox; Figure S6: MS/MS spectra and fragmentation patterns for 4CEC and 4CECox; Figure S7: MS/MS spectra and fragmentation patterns for 3CMC and 3CMCox; Figure S8: Graphs obtained for the PLS models; Table S1: Ionization and fragmentation parameters; Table S2: Gradient employed for UPLC separation; Table S3: Oxidation peaks for single solutions of synthetic cathinones; Table S4: Oxidation peaks for single solutions of adulterants/cutting agents; Table S5: Major fragmentation products obtained for PVP and its oxidation product; Table S6: Major fragmentation products obtained for NEH and its oxidation product; Table S7: Major fragmentation products obtained for 4CEC and its oxidation product; Table S8: Major fragmentation products obtained for 3CMC and its oxidation product; Table S9: Peak potentials obtained for equimolar binary mixtures; Table S10: Current intensities obtained for equimolar binary mixtures; Table S11: The objectives set for the optimization study; Table S12: The objectives set for the screening study; Table S13: The results obtained for the analysis of seized samples.
