# Supplementary material for: Forensic Analysis of Synthetic Cathinones on Nanomaterials-Based Platforms: Chemometric-Assisted Voltametric and UPLC-MS/MS Investigation
Source: Nanomaterials (Basel). 2023 Aug 22;13(17):2393. doi: 10.3390/nano13172393 (PMC10489959; doi:10.3390/nano13172393)
Supplement: Supplementary file 1 [file nanomaterials-13-02393-s001.zip › nanomaterials-2548518-supplementary.pdf]

Supplementary material

Forensic Analysis of Synthetic Cathinones on Nanomaterials-based Platforms: Chemometric-Assisted Voltametric and UPLC - MS/MS Investigation

Ana-Maria Dragan [1,2], Bogdan Feier [1], Mihaela Tertiș [1], Ede Bodoki [1,*], Florina Truta [1], Maria-Georgia Ștefan [3], Béla Kiss [3], Filip Van Durme [4], Karolien De Wael [2,5], Radu Oprean [1], Cecilia Cristea [1,*]

[1] Department of Analytical Chemistry, Faculty of Pharmacy, 'Iuliu Hațieganu' University of Medicine and Pharmacy, Pasteur 6, 400349 Cluj-Napoca, Romania; Ana.Dragan@umfcluj.ro; Feier.George@umfcluj.ro; Mihaela.Tertis@umfcluj.ro; BodokiE@umfcluj.ro; Florina.Truta@umfcluj.ro; ROprean@umfcluj.ro; CCristea@umfcluj.ro
[2] A-Sense Lab, University of Antwerp, Groenenborgerlaan 171, 2010 Antwerp, Belgium; Ana-Maria.Dragan@student.uantwerpen.be; Karolien.DeWael@uantwerpen.be
[3] Department of Toxicology, Faculty of Pharmacy, 'Iuliu Hațieganu' University of Medicine and Pharmacy, Pasteur 6, 400349 Cluj-Napoca, Romania; Stefan.Georgia@umfcluj.ro; KBela@umfcluj.ro;
[4] Drugs and Toxicology Department, National Institute for Criminalistics and Criminology (NICC), Vilvoordsesteenweg 100, 1120, Brussels, Belgium; Filip.VanDurme@just.fgov.be
[5] NANOlab Center of Excellence, University of Antwerp, Groenenborgerlaan 171, 2010 Antwerp, Belgium; Karolien.DeWael@uantwerpen.be
* Correspondence: CCristea@umfcluj.ro; Tel.: +40 721 375 789 (C.C.); BodokiE@umfcluj.ro (E.B.).

Table of contents

Figures …… **SM-2**
Figure S1. Baseline-corrected SWVs of adulterants / cutting agents …… SM-2
Figure S2. Chemical structures of the adulterants / cutting agents …… SM-2
Figure S3. Overlapped SWVs of SCs and adulterants / cutting agents …… SM-3
Figure S4. MS/MS spectra and fragmentation patterns for PVP and PVPox …… SM-4
Figure S5. MS/MS spectra and fragmentation patterns for NEH and NEHox …… SM-5
Figure S6. MS/MS spectra and fragmentation patterns for 4CEC and 4CECox …… SM-6
Figure S7. MS/MS spectra and fragmentation patterns for 3CMC and 3CMCox …… SM-7
Figure S8. Graphs obtained for the PLS models …… SM-8

Tables …… **SM-9**
Table S1. Ionization and fragmentation parameters …… SM-9
Table S2. Gradient employed for UPLC separation …… SM-9
Table S3. Oxidation peaks for single solutions of synthetic cathinones …… SM-10
Table S4. Oxidation peaks for single solutions of adulterants / cutting agents …… SM-12
Table S5. Major fragmentation products obtained for PVP and its oxidation product …… SM-13
Table S6. Major fragmentation products obtained for NEH and its oxidation product …… SM-14
Table S7. Major fragmentation products obtained for 4CEC and its oxidation product …… SM-15
Table S8. Major fragmentation products obtained for 3CMC and its oxidation product …… SM-16
Table S9. Peak potentials obtained for equimolar binary mixtures …… SM-17
Table S10. Current intensities obtained for equimolar binary mixtures …… SM-18
Table S11. The objectives set for the optimisation study …… SM-19
Table S12. The objectives set for the screening study …… SM-19
Table S13. The results obtained for the analysis of seized samples …… SM-20

Figures

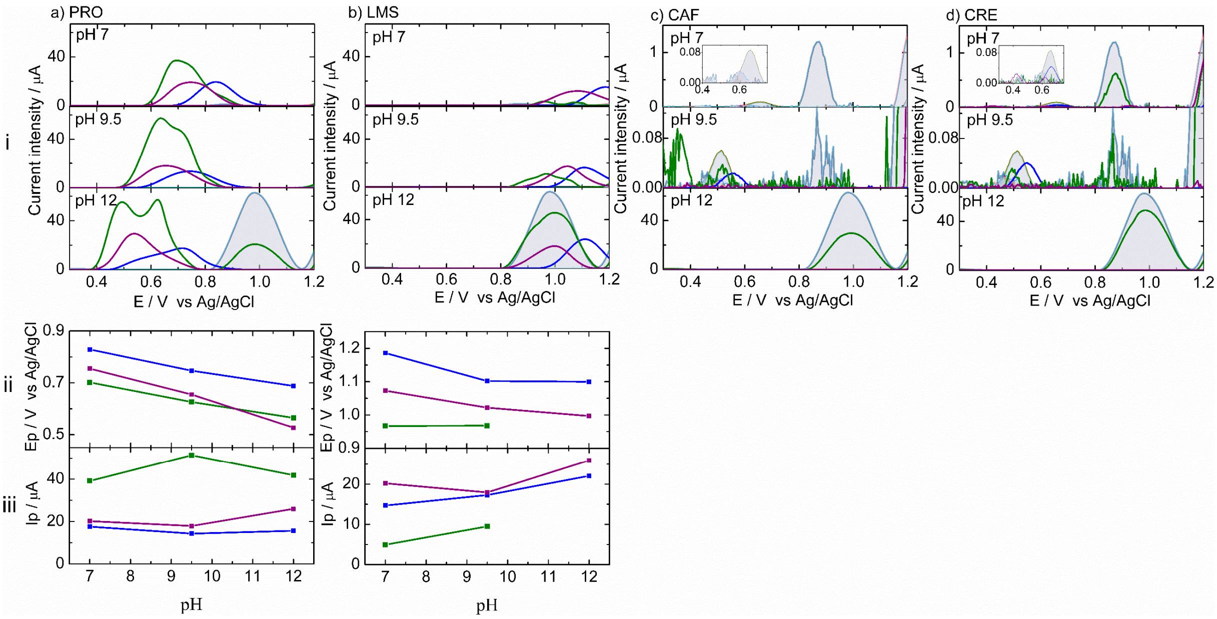

Figure S1. Electrochemical characterization of the four adulterants/ cutting agents: procaine – PRO (a), levamisole – LMS (b), caffeine – CAF (c), and creatine – CRE (d), on G-SPEs (blue line/squares), GPH-SPEs (green line/squares) and MWCNT-SPEs (purple line/squares) in 0.5 mM solutions in PBS 20 mM at pH 7, pH 9.5 and pH 12: baseline corrected square wave voltammograms (i); the shift of the peak potential (Ep; ii) and the Ip (Ip, iii) with the pH of the electrolyte for PRO and LMS, respectively. The blanks are represented with blue/green/purple lines and grey fill.

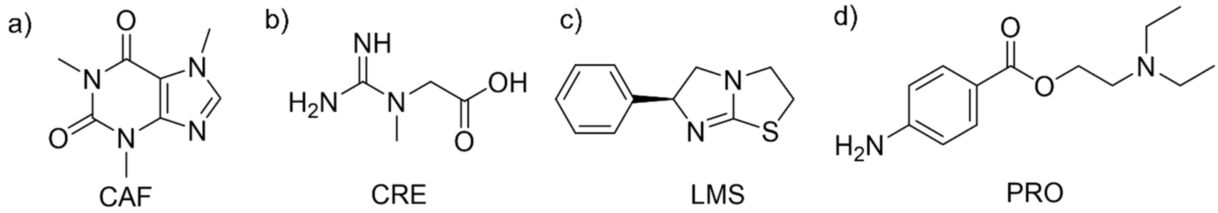

Figure S2. Structures of the adulterants/cutting agents used in the study: a) caffeine – CAF, b) creatine – CRE, c) levamisole – LMS and d) procaine - PRO.

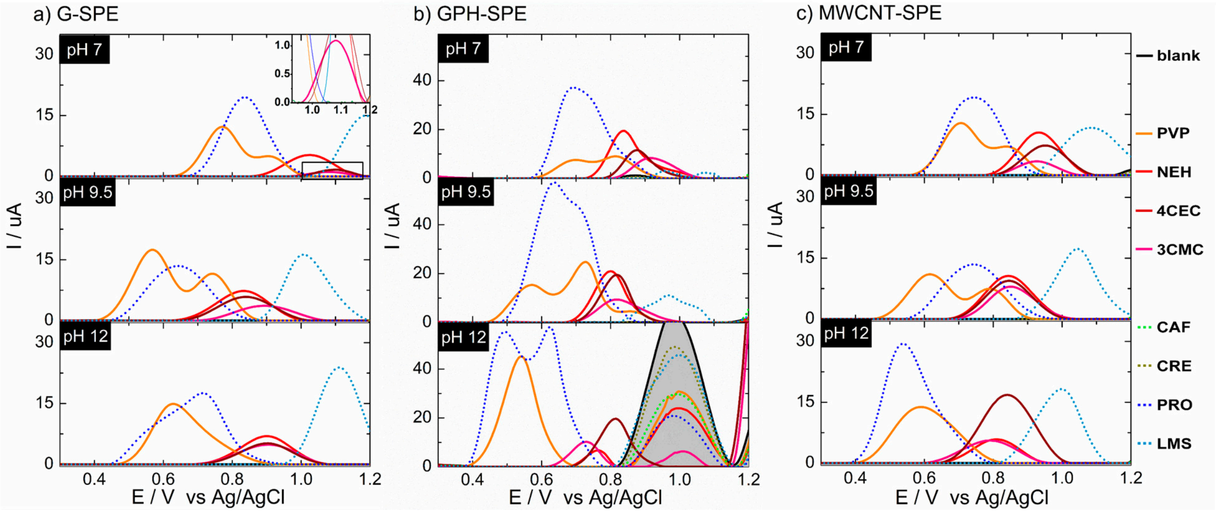

Figure S3. Baseline corrected square wave voltammograms of the four cathinones (α - pyrrolidinovalerophenone - PVP - orange line, *N*-ethylhexedrone - NEH - red line, 4-chloroethcathinone - 4CEC - dark red line, and 3-chloromethcathione - 3CMC - pink line) and the four adulterants/cutting agents (caffeine - CAF - light green dots, creatine - CRE - dark yellow dots, procaine - PRO - blue dots and levamisole - LMS - light blue dots) analyzed in 0.5 mM solutions in PBS pH 7, pH 9.5 and pH 12, on different platforms: a) graphite screen-printed electrode (G-SPEs, b) graphene-SPE (GPH-SPE) and (c) multi-walled carbon nanotubes-SPE (MWCNT-SPE). The grey areas represent the blanks obtained for each set of conditions.

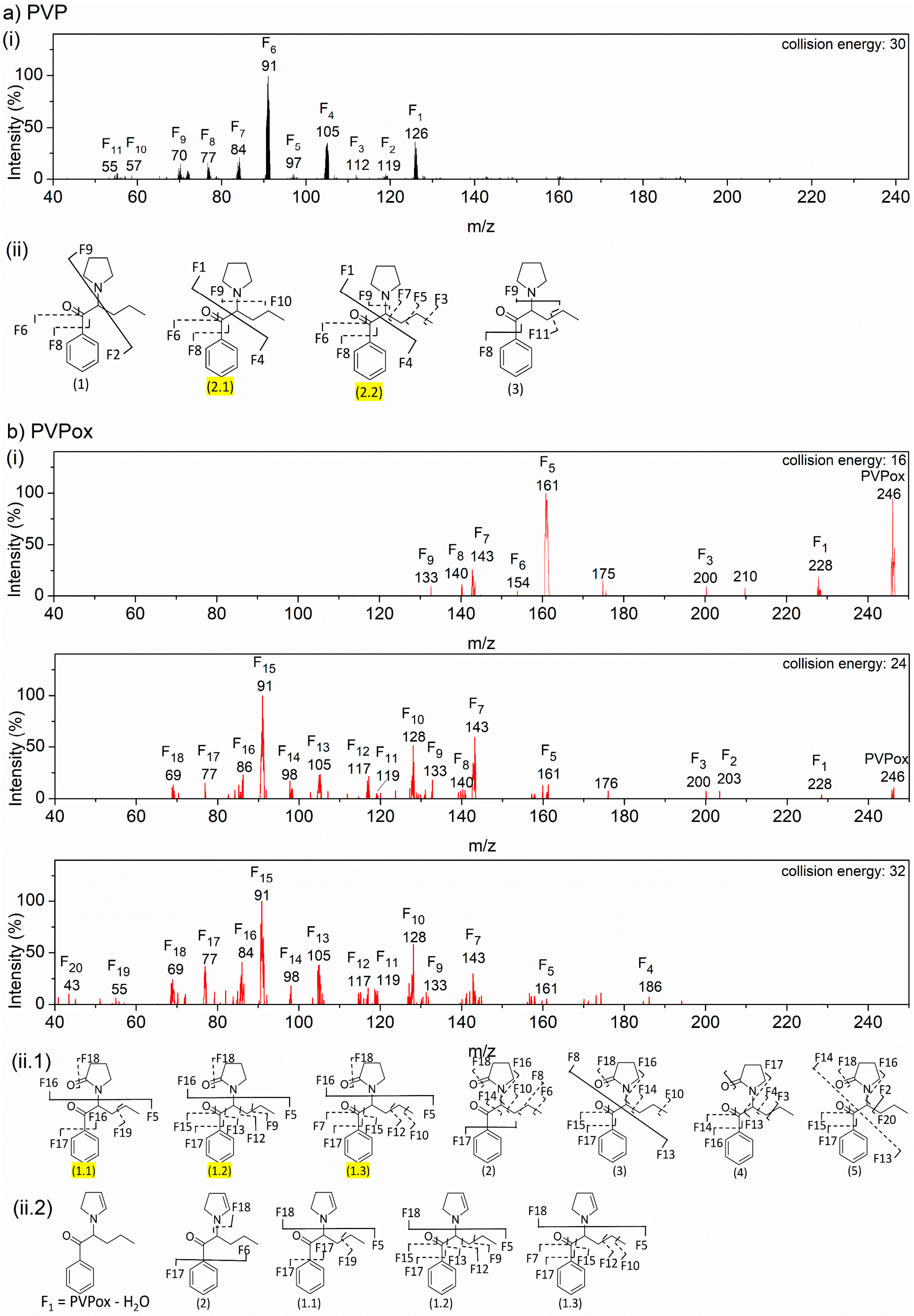

Figure S4. MS/MS spectra (at various collision energies) (i) and the schematic representation of the fragmentation patterns without (ii.1) and with (ii.2) a water loss step proposed for α - pyrrolidinovalerophenone (PVP, a) and for it's oxidation product (PVPox, b). Highlight: main fragmentation patterns.

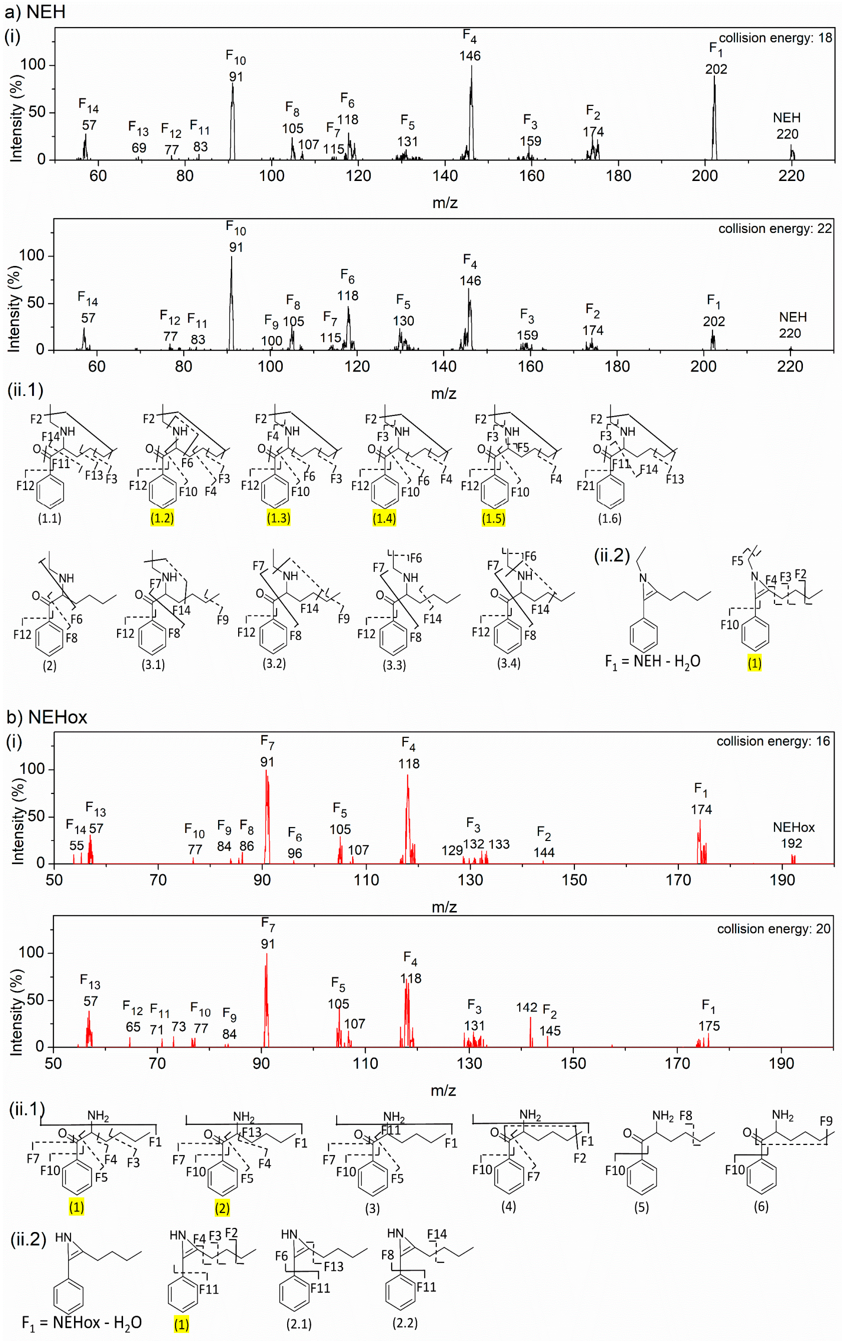

Figure S5. MS/MS spectra (at various collision energies) (i) and the schematic representation of the fragmentation patterns without (ii.1) and with (ii.2) a water loss step proposed for *N*-ethylhexedrone (NEH, a) and its oxidation product (NEHox, b). Highlight: main fragmentation patterns.

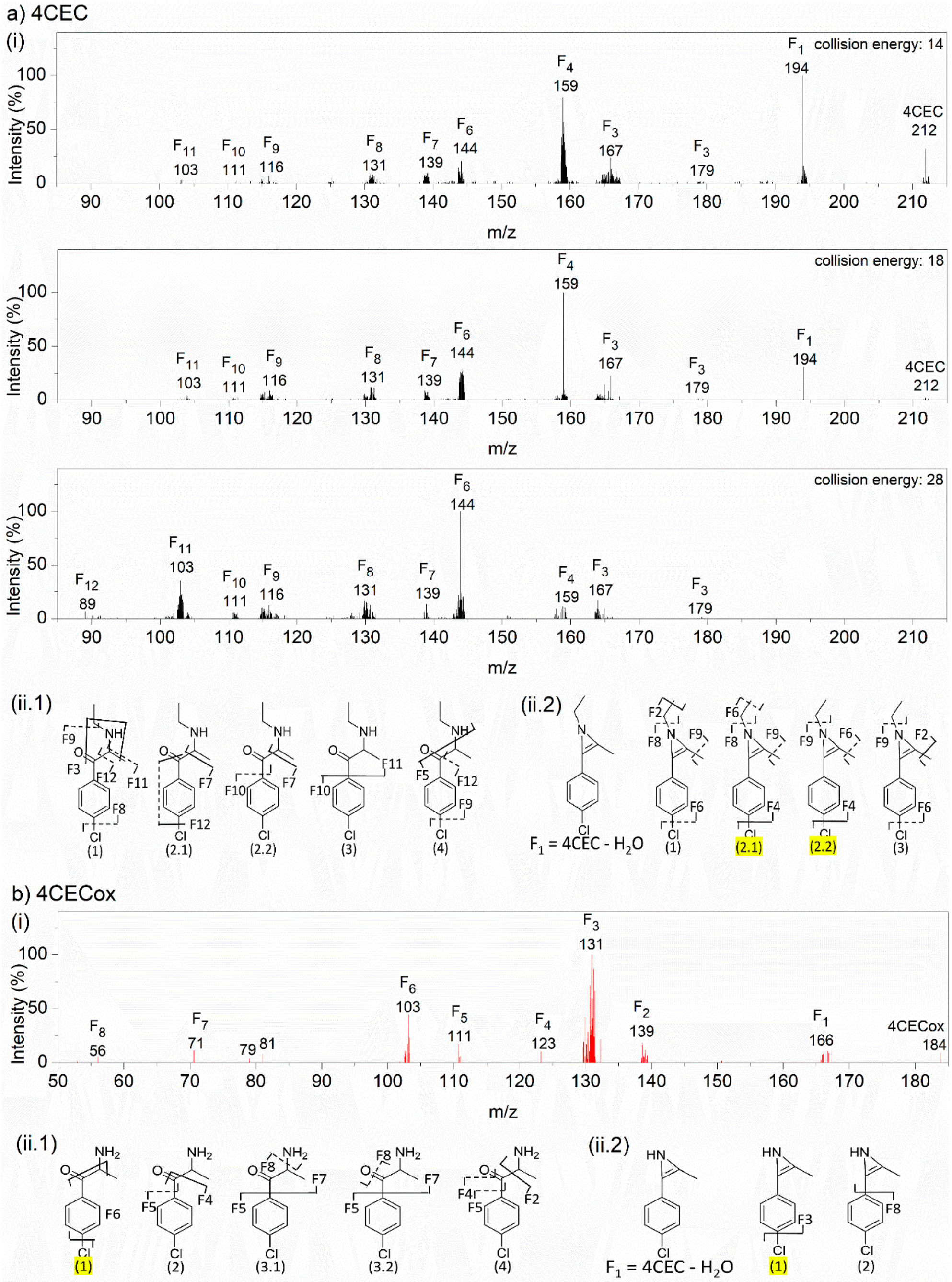

Figure S6. MS/MS spectra (at various collision energies) (i) and the schematic representation of the fragmentation patterns without (ii.1) and with (ii.2) a water loss step proposed for 4-chloroethcathinone (4CEC, a) and its oxidation product (4CECox, b). Highlight: main fragmentation patterns.

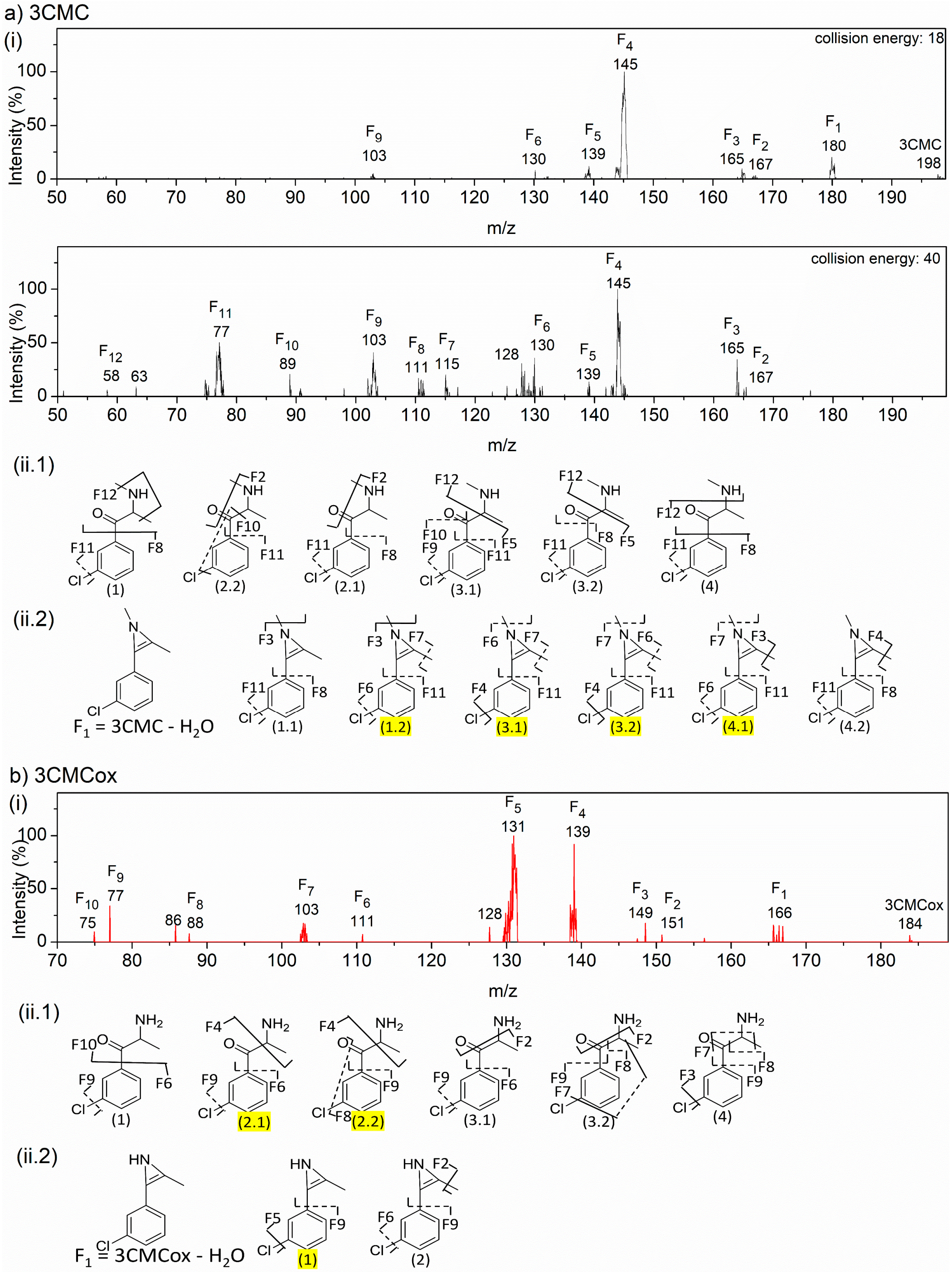

Figure S7. MS/MS spectra (at various collision energies) (i) and the schematic representation of the fragmentation patterns without (ii.1) and with (ii.2) a water loss step proposed for 3-chloromethcathione (3CMC, a) and its oxidation product (3CMCox, b). Highlight: main fragmentation patterns.

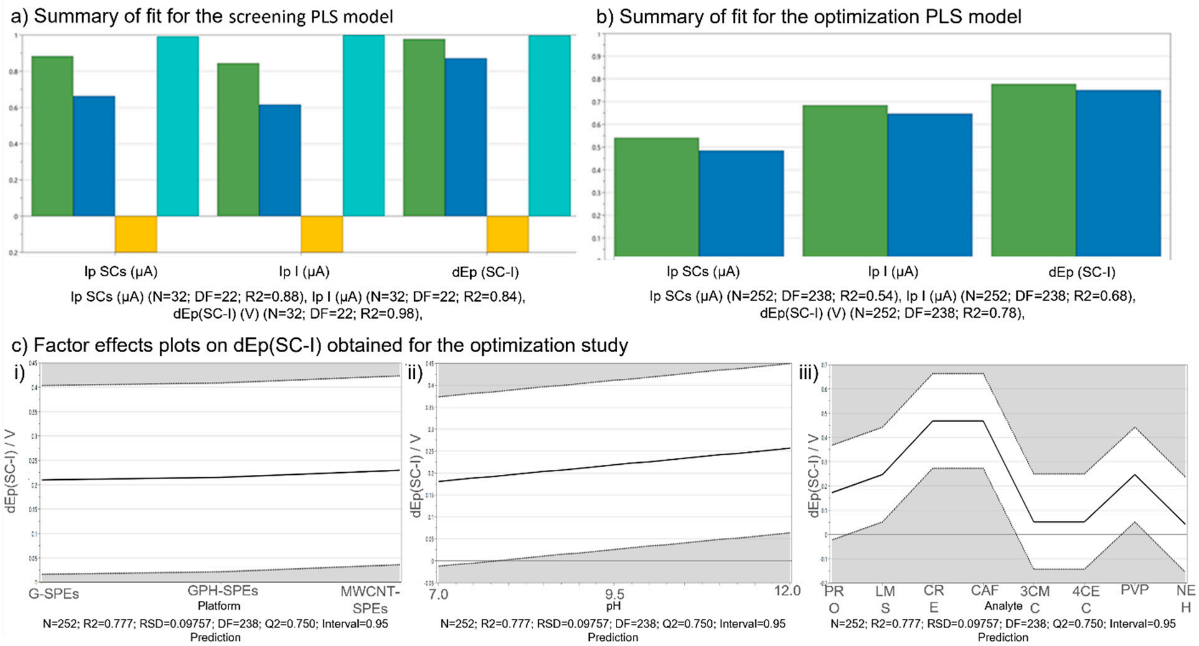

Figure S8. Representation of the summaries of fit obtained for the screening (a) and optimization (b) PLS models and the factor effects plots obtained for the optimisation study on the platform (bi), pH (bii) and analyte (biii). Green columns: R2 (goodness of fit); Blue columns: Q2 (predictive power); Yellow columns: model validity; Cyan columns: reproducibility; dE_p: peak potential difference; I_p: peak current; I: interferent; SCs: synthetic cathinones.

Tables

Table S1. Ionization and fragmentation parameters

Synthetic cathinone	Con	Collision energy
PVP	42	30
PVPox	32	16, 24, 32
NEH	28	18, 22
NEHox	30	16, 20
3CMC	30	18, 40
3CMCox	ramp	Ramp
4CEC	30	14, 18, 28
4CECox	ramp	Ramp

3CMC: 3-chloromethcathione; **4CEC**: 4-chloroethcathinone; **NEH**: *N*-ethylhexedrone; **ox**: oxidation product; **PVP**: α - pyrrolidinovalerophenone

Table S2. Gradient employed for UPLC separation

Time (min)	Flow (mL/min)	%A	%B
0.0	0.50	98	2
0.5	0.50	98	2
2.0	0.50	60	40
2.2	0.50	60	40
3.7	0.50	10	90
4.0	0.50	0	100
5.5	0.50	98	2
7.5	0.50	98	2

A: 5 mM ammonium acetate in water, containing 0.1 % formic acid; **B:** 0.1 % formic acid in methanol.

Table S3. Peak potential values and current intensities of the oxidation peaks obtained for the SCs by SWV at pH 7, pH 9.5 and pH 12 on G-SPE, GPH-SPE and MWCNT-SPE for the reproducibility study on the three platforms and at three pH values

No.	SC	pH	SPE	Ep (V)		Ip (μA)	
				Average	RSD (%)	Average	RSD (%)
1	PVP	7.0	G	0.77 (0.76/0.92)*	0.31 (0.31/0.26)*	11.74 (9.03/2.04)*	6.71 (8.07/6.50)*
2	PVP	7.0	GPH	0.82 (0.69/0.83)*	0.29 (0.34/0.00)*	9.51 (3.90/5.04)*	3.77 (12.05/4.25)*
3	PVP	7.0	MWCNT	0.71 (0.70/0.86)*	1.45 (1.17/0.95)*	13.61 (9.35/2.31)*	19.66 (16.59/8.68)*
4	PVP	9.5	G	0.67 (0.66/0.85)*	1.23 (1.55/0.96)*	14.09 (11.01/6.17)*	16.87 (13.18/10.88)*
5	PVP	9.5	GPH	0.74 (0.57/0.73)*	2.09 (1.90/0.32)*	20.76 (8.24/15.42)*	19.34 (5.56/7.52)*
6	PVP	9.5	MWCNT	0.62 (0.61/0.80)*	1.14 (0.85/0.27)*	12.87 (9.30/4.83)*	17.95 (15.23/6.61)*
7	PVP	12.0	G	0.64	3.03	18.35	13.66
8	PVP	12.0	GPH	0.54	0.83	47.33	3.77
9	PVP	12.0	MWCNT	0.60	0.79	15.84	10.69
10	NEH	7.0	G	1.04	0.84	3.07	3.99
11	NEH	7.0	GPH	0.85	1.17	17.66	14.84
12	NEH	7.0	MWCNT	0.92	0.51	10.24	11.35
13	NEH	9.5	G	0.94	2.00	7.77	10.06
14	NEH	9.5	GPH	0.79	2.09	21.52	1.89
15	NEH	9.5	MWCNT	0.84	0.84	10.42	9.66
16	NEH	12.0	G	0.90	0.53	6.55	7.69
17	NEH	12.0	GPH	0.77	0.31	7.43	11.65
18	NEH	12.0	MWCNT	0.80	0.78	5.59	11.87
19	4CEC	7.0	G	1.10	0.43	1.64	14.98
20	4CEC	7.0	GPH	0.87	0.27	11.51	6.18
21	4CEC	7.0	MWCNT	0.85	0.48	17.25	1.74
22	4CEC	9.5	G	0.96	1.61	4.93	12.83
23	4CEC	9.5	GPH	0.82	1.65	18.71	4.72
24	4CEC	9.5	MWCNT	0.84	0.28	9.37	5.29
25	4CEC	12.0	G	0.94	2.80	4.49	11.05
26	4CEC	12.0	GPH	0.82	0.86	19.96	1.62
27	4CEC	12.0	MWCNT	0.85	0.48	17.25	1.74
28	3CMC	7.0	G	1.08	0.22	1.22	7.73
29	3CMC	7.0	GPH	0.93	1.34	7.99	3.49
30	3CMC	7.0	MWCNT	0.92	0.25	3.48	7.07
31	3CMC	9.5	G	0.99	0.48	3.54	3.40
32	3CMC	9.5	GPH	0.82	0.58	11.26	15.92
33	3CMC	9.5	MWCNT	0.85	0.28	7.90	0.72
34	3CMC	12.0	G	0.90	1.68	4.70	2.61

Table S3. Peak potential values and current intensities of the oxidation peaks obtained for the SCs by SWV at pH 7, pH 9.5 and pH 12 on G-SPE, GPH-SPE and MWCNT-SPE for the reproducibility study on the three platforms and at three pH values

No.	SC	pH	SPE	Ep (V)		Ip (μA)	
				Average	RSD (%)	Average	RSD (%)
35	3CMC	12.0	GPH	0.73	0.00	10.40	2.11
36	3CMC	12.0	MWCNT	0.78	0.60	5.87	7.96

*values for the two incomplete separated peaks (P1 and P2)

3CMC: 3-chloromethcathione; **4CEC**: 4-chloroethcathinone; **E_p**: peak potential; **G**: graphite; **GPH**: graphene; **I_p**: peak current; **MWCNTs**: multi-walled carbon nanotubes; **NEH**: *N*-ethylhexedrone; **PVP**: α-pyrrolidinovalerophenone; **SPE**: screen-printed electrode.

Table S4. Peak potential values and current intensities of the oxidation peaks obtained for the adulterants / cutting agents by SWV at pH 7, pH 9.5 and pH 12 on G-SPE, GPH-SPE and MWCNT-SPE for the reproducibility study on the three platforms and at three pH values

No.	Adulterant	pH	SPE	Ep (V)		Ip (μA)	
				Average	RSD (%)	Average	RSD
1	PRO	7.0	G	0.829	0.57	17.60	8.80
2	PRO	7.0	GPH	0.702	1.21	39.13	11.49
3	PRO	7.0	MWCNT	0.755	1.43	20.19	8.60
4	PRO	9.5	G	0.747	0.32	14.32	4.99
5	PRO	9.5	GPH	0.626	1.00	51.37	8.25
6	PRO	9.5	MWCNT	0.655	0.62	17.93	0.66
7	PRO	12.0	G	0.688	3.27	15.64	11.64
8	PRO	12.0	GPH	0.565	11.69	41.83	36.13
9	PRO	12.0	MWCNT	0.527	1.61	25.95	9.54
10	LMS	7.0	G	1.186	0.36	14.68	1.43
11	LMS	7.0	GPH	0.967	0.42	4.93	35.40
12	LMS	7.0	MWCNT	1.073	2.26	10.95	17.25
13	LMS	9.5	G	1.102	0.35	17.27	7.63
14	LMS	9.5	GPH	0.968	0.22	9.52	13.17
15	LMS	9.5	MWCNT	1.022	1.71	12.41	27.99
16	LMS	12.0	G	1.100	1.29	22.07	5.78
17	LMS	12.0	GPH	-	-	-	-
18	LMS	12.0	MWCNT	0.997	0.00	18.73	2.71
19	CRE	7.0	G	-	-	-	-
20	CRE	7.0	GPH	-	-	-	-
21	CRE	7.0	MWCNT	-	-	-	-
22	CRE	9.5	G	-	-	-	-
23	CRE	9.5	GPH	-	-	-	-
24	CRE	9.5	MWCNT	-	-	-	-
25	CRE	12.0	G	-	-	-	-
26	CRE	12.0	GPH	-	-	-	-
27	CRE	12.0	MWCNT	-	-	-	-
28	CAF	7.0	G	-	-	-	-
29	CAF	7.0	GPH	-	-	-	-
30	CAF	7.0	MWCNT	-	-	-	-
31	CAF	9.5	G	-	-	-	-
32	CAF	9.5	GPH	-	-	-	-
33	CAF	9.5	MWCNT	-	-	-	-
34	CAF	12.0	G	-	-	-	-
35	CAF	12.0	GPH	-	-	-	-
36	CAF	12.0	MWCNT	-	-	-	-

CAF: caffeine; **CRE**: creatine; **E_p**: peak potential; **G**: graphite; **GPH**: graphene; **I_p**: peak current; **LMS**: levamisole; **MWCNTs**: multi-walled carbon nanotubes; **PRO**: procaine; **SPE**: screen-printed electrode.

Table S5. Major fragmentation products obtained for PVP (a) and its oxidation product (PVPox; b)

(a) Compound	PVP	F6	F1	F4
Measured [*m/z*]	232	91	126	105
Theoretical [*m/z*]	232	89	127	105
Chemical formula	$C_{15}H_{21}ON$	C_7H_5	$C_8H_{16}N$	C_7H_5O
Structure	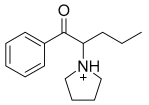	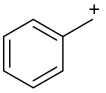	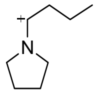	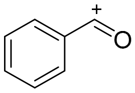
(b) Compound	PVPox	F5	F15	F10
Measured [*m/z*]	246	161	91	128
Theoretical [*m/z*]	246	162	89	130
Chemical formula	$C_{15}H_{19}O_2N$	$C_{11}H_{13}O$	C_7H_5	$C_{10}H_{10}$
Structure	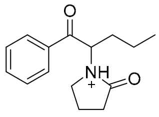	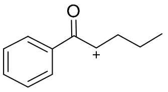	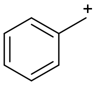	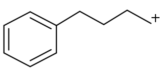

PVP: α-pyrrolidinovalerophenone; **F**: fragment.

Table S6. Major fragmentation products obtained for NEH (a) and its oxidation product (NEHox; b)

(a)	Compound	NEH	F1	F4	F10
	Measured [*m/z*]	220	202	146	91
	Theoretical [*m/z*]	220	202	146	89
	Chemical formula	$C_{14}H_{21}ON$	$C_{14}H_{19}N$	$C_{10}H_{11}N$	C_7H_5
	Structure	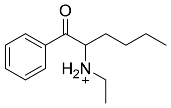	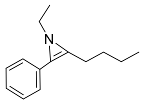	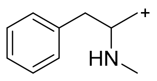	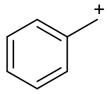
(b)	Compound	NEHox	F7	F4	F1
	Measured [*m/z*]	192	91	118	174
	Theoretical [*m/z*]	192	89	118	174
	Chemical formula	$C_{12}H_{17}ON$	C_7H_5	C_8H_6O	$C_{12}H_{15}N$
	Structure	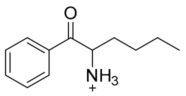	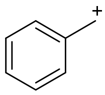	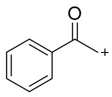	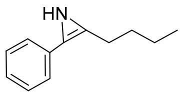

NEH: *N*-ethylhexedrone; **F**: fragment.

Table S7. Major fragmentation products obtained for 4CEC (a) and its oxidation product (4CECox; b)

(a)	Compound	4CEC	F1	F4	F6
	Measured [*m*/*z*]	212	194	159	144
	Theoretical [*m*/*z*]	212	194	159	144
	Chemical formula	$C_{11}H_{14}ONCl$	$C_{11}H_{12}NCl$	$C_{11}H_{12}N$	$C_{10}H_{9}N$
	Structure	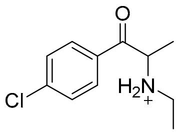	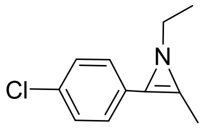	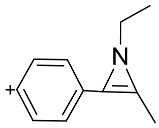	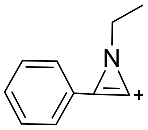
(b)	Compound	4CECox	F3	F6	
	Measured [*m*/*z*]	184	131	103	
	Theoretical [*m*/*z*]	184	131	102	
	Chemical formula	$C_{9}H_{10}ONCl$	$C_{9}H_{8}N$	$C_{8}H_{5}$	
	Structure	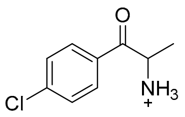	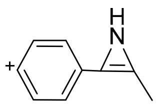	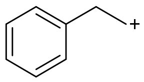	

4CEC: 4-chloroethcathinone; **F**: fragment.

Table S8. Major fragmentation products obtained for 3CMC (a) and it's oxidation product (3CMCox; b)

(a)	Compound	3CMC	F4	F3	F11
	Measured [*m*/*z*]	198	145	165	77
	Theoretical [*m*/*z*]	198	145	165	77
	Chemical formula	$C_{10}H_{12}ONCl$	$C_{10}H_{10}N$	C_9H_7NCl	C_6H_4
	Structure	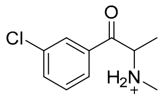	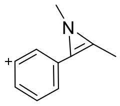	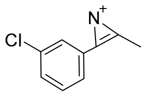	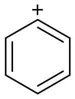
(b)	Compound	3CMCox	F5	F4	
	Measured [*m*/*z*]	184	131	139	
	Theoretical [*m*/*z*]	184	131	140	
	Chemical formula	$C_9H_{10}ONCl$	C_9H_8N	C_7H_4OCl	
	Structure	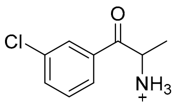	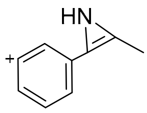	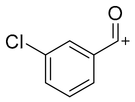	

3CMC: 3-chloromethcathione; **F**: fragment.

Table S9. Peak potential values of the oxidation peaks obtained by SWV at pH 7, pH 9.5 and pH 12 on G-SPE, GPH-SPE and MWCNT-SPE for the equimolar binary mixtures of cathinones and adulterants included in the study according to the experimental design

**Run Order**	**SCs**	**A**	**pH**	**SPE**	**Ep (V)**			
					**Cathinone (SCs)**		**Adulterant (A)**	
					**Average**	**RSD%**	**Average**	**RSD%**
1	3CMC	PRO	7	graphite	-	-	0.827	0.415
2	3CMC	LMS	12	MWCNT	-	-	1.010	0.865
3	4CEC	PRO	9.5	MWCNT	0.957	0.526	0.643	0.411
4	3CMC	CAF	7	GPH	-	-	-	-
5	3CMC	CAF	7	GPH	-	-	-	-
6	4CEC	PRO	9.5	MWCNT	0.957	0.060	0.641	0.360
7	4CEC	PRO	9.5	MWCNT	0.955	0.363	0.640	0.090
8	3CMC	PRO	7	graphite	-	-	0.824	0.321
9	NEH	CRE	7	MWCNT	0.921	0.543	-	-
10	3CMC	LMS	12	MWCNT	-	-	1.014	0.285
11	PVP	CAF	9.5	MWCNT	0.624	0.801	-	-
12	NEH	PRO	12	GPH	0.748	0.473	0.497	1.627
13	4CEC	CAF	12	graphite	0.916	1.692	-	-
14	NEH	CRE	7	MWCNT	0.925	0.348	-	-
15	NEH	LMS	9.5	graphite	-	-	1.098	0.911
16	4CEC	LMS	7	GPH	0.85	0.59	1.026	5.805
17	3CMC	CAF	7	GPH	-	-	-	-
18	PVP	LMS	7	graphite	0.758	1.523	1.199	0.626
19	4CEC	PRO	9.5	MWCNT	0.962	0.000	0.644	0.781
20	3CMC	CAF	7	GPH	-	-	-	-
21	NEH	PRO	12	GPH	0.753	1.014	0.491	0.588
22	PVP	CAF	9.5	MWCNT	0.619	0.000	-	-
23	3CMC	CRE	9.5	graphite	0.980	0.294	-	-
24	4CEC	CAF	12	graphite	0.909	1.384	-	-
25	PVP	LMS	7	graphite	0.760	0.000	1.201	0.240
26	4CEC	CAF	12	graphite	0.913	1.265	-	-
27	4CEC	CAF	12	graphite	0.909	0.317	-	-
28	3CMC	CRE	9.5	graphite	0.955	2.634	-	-
29	4CEC	LMS	7	GPH	0.851	0.000	0.962	0.52
30	PVP	CRE	12	GPH	0.529	0.945	-	-
31	PVP	CRE	12	GPH	0.514	2.574	-	-
32	NEH	LMS	9.5	graphite	-	-	1.098	0.455

3CMC: 3-chloromethcathione; **4CEC**: 4-chloroethcathinone; **CAF**: caffeine; **CRE**: creatine; **E_p**: peak potential; **G**: graphite; **GPH**: graphene; **LMS**: levamisole; **MWCNTs**: multi-walled carbon nanotubes; **NEH**: *N*-ethylhexedrone; **PRO**: procaine; **PVP**: α-pyrrolidinovalerophenone; **SPE**: screen-printed electrode

Table S10. Current intensities (Ip) of the oxidation peaks obtained by SWV at pH 7, pH 9.5 and pH 12 on G-SPE, GPH-SPE and MWCNT-SPE for the equimolar binary mixtures of the synthetic cathinones (SCs) and adulterants (A) included in the study according to the experimental design

Run Order	SCs	A	pH	Electrode	Ip (μA)			
					Cathinone (SCs)		Adulterant (A)	
					Average	RSD%	Average	RSD%
1	3CMC	PRO	7	graphite	-	-	18.43	1.82
2	3CMC	LMS	12	MWCNT	-	-	15.23	5.07
3	4CEC	PRO	9.5	MWCNT	1.04	24.81	17.52	4.83
4	3CMC	CAF	7	GPH	-	-	-	-
5	3CMC	CAF	7	GPH	-	-	-	-
6	4CEC	PRO	9.5	MWCNT	1.03	5.92	17.44	2.38
7	4CEC	PRO	9.5	MWCNT	1.19	36.31	18.10	3.73
8	3CMC	PRO	7	graphite	-	-	18.35	2.29
9	NEH	CRE	7	MWCNT	9.40	5.64	-	-
10	3CMC	LMS	12	MWCNT	-	-	15.42	0.73
11	PVP	CAF	9.5	MWCNT	11.54	1.04	-	-
12	NEH	PRO	12	GPH	0.27	82.72	46.12	5.89
13	4CEC	CAF	12	graphite	2.34	40.45	-	-
14	NEH	CRE	7	MWCNT	9.32	1.12	-	-
15	NEH	LMS	9.5	graphite	-	-	11.11	33.42
16	4CEC	LMS	7	GPH	2.22	18.27	3.68	55.83
17	3CMC	CAF	7	GPH	-	-	-	-
18	PVP	LMS	7	graphite	10.45	5.18	14.47	15.53
19	4CEC	PRO	9.5	MWCNT	1.07	36.19	17.68	4.12
20	3CMC	CAF	7	GPH	-	-	-	-
21	NEH	PRO	12	GPH	0.44	70.45	44.81	4.12
22	PVP	CAF	9.5	MWCNT	13.93	34.71	-	-
23	3CMC	CRE	9.5	graphite	0.81	19.43	-	-
24	4CEC	CAF	12	graphite	3.24	39.66	-	-
25	PVP	LMS	7	graphite	10.30	7.01	14.16	14.43
26	4CEC	CAF	12	graphite	3.25	41.16	-	-
27	4CEC	CAF	12	graphite	3.11	45.78	-	-
28	3CMC	CRE	9.5	graphite	1.20	33.00	-	-
29	4CEC	LMS	7	GPH	2.67	11.12	2.59	71.01
30	PVP	CRE	12	GPH	44.76	9.19	-	-
31	PVP	CRE	12	GPH	39.93	23.20	-	-
32	NEH	LMS	9.5	graphite	-	-	10.48	19.23

3CMC: 3-chloromethcathione; **4CEC**: 4-chloroethcathinone; **CAF**: caffeine; **CRE**: creatine; **G**: graphite; **GPH**: graphene; **I_p**: peak current; **LMS**: levamisole; **MWCNTs**: multi-walled carbon nanotubes; **NEH**: *N*-ethylhexedrone; **PRO**: procaine; **PVP**: α-pyrrolidinovalerophenone; **SPE**: screen-printed electrode

Table S11. The objectives set for the screening study on the binary mixtures using PLS

Variable	Condition	Objective	Min	Target	Max	Predicted min	Predicted max	Desirability type	Desirability weight
Ip SCs (μA)	Desired	Maximize	5	90.5	100	13.6314	36.2476	Limit	1
Ip A (μA)	Desired	Minimize	3	4.7	20	-9.17253	26.1057	Limit	0.5
dEp(SC-A) (V)	Required	Maximize	0.1	0.91	1	0.510482	0.85247	Limit	1

A: adulterant; **dEp(SC-A)**: difference between the peak potential obtained for the SC and the adulterant in the mixture; **Ip**: current intensity; **SC**: synthetic cathinone.

Table S12. The objectives set for the optimisation study on the single solutions using PLS

Variable	Condition	Objective	Min	Target	Max	Predicted min	Predicted max	Desirability type	Desirability weight
Ip SCs (μA)	Desired	Maximize	5	90.5	100	3.66168	16.884	Limit	1
Ip I (μA)	Desired	Minimize	3	4.7	20	-3.76087	32.3066	Limit	0.5
dEp(SC-I) (V)	Required	Inside	0.1	0.1	1	0.04671	0.47623	Limit	1

dEp: difference between the peak potential obtained for each SC and the I; **I**: interferent; **Ip**: current intensity; **SC**: synthetic cathinone.

Table S13. Results of the confiscated samples assessment using different analytical techniques

Sample	Sample aspect	GC-MS	EC device (Ep / V)	Raman
S1	-	CMC	0.78	-
S2	white powder	CMC	0.25; 0.61; 0.85	cathinone
S3	white powder	FMC	0.91	cathinone
S4	white powder	FMC	0.29; 0.92	4Cl-aPVP
S5	white powder	MMC	0.95	3MMC
S6	white powder	MMC	0.40; 0.79; 1.03	3MMC
S7	white powder	ETC	0.88	cathinone
S8	white powder	MBP	0.27; 0.86	PVP
S9	white powder	aPHiP	0.61	PVP
S10	white powder	COC, LMS	0.09; 0.92	cocaine mix
S11	pink powder	MDMA	0.26; 0.64; 0.99	unknown
S12	yellow powder	AM, CAF	0.85	caffeine
S13	brown powder	HER, CAF, PARA, 6-MAM, NOSC, PAPAV	0.14; 0.58; 0.92	no identification
S14	white powder	MA, DMS	0.18; 0.87	cocaine mix
S15	transparent liquid	GBL, GHB	0.57; 0.91	no identification
S16	brown powder	CAF	0.27; 0.66; 0.92	error
S17	red liquid	CAF	0.37	unknown
S18	orange powder	DMT	0.36; 0.92	oleic acid
S19	white powder	ANRC	0.23; 0.90	anise seed oil
S20	white powder	nicotine	0.86	nifoxipam
S21	white powder	NSI-189	0.03; 0.92	PPDB

4Cl-aPVP: 4-chlor-alpha-pyrrolidinovalerophenone; **6-MAM**: 6-monoacetylmorphine; **AM**: amphetamine; **ANRC**: aniracetam; **aPHiP**: α-Pyrrolidinohexiophenone; **CMC**: chloromethcathinone; **CAF**: caffeine; **COC**: cocaine; **DMS**: dimethyl sulfone; **DMT**: dimethyltryptamine; **EC**: electrochemical; **ETC**: ethcathinone; **FMC**: fluoromethcathinone; **GBL**: gamma-butyrolactone; **GHB**: gamma hydroxybutyrate; **HER**: heroine; **LMS**: levamisole; **MA**: methamphetamine; **MBP**: methylbuphedrone; **MDMA**: 3,4 -methylenedioxymethamphetamine; **MMC**: methylmethcathione; **NOSC**: noscapine; **NSI-189**: a novel neurogenic compound; **PAPAV**: papaverine; **PARA**: paracetamol.
